# Molecular cloning and functional characterization of the promoter of a novel *Aspergillus flavus* inducible gene (*AhOMT1*) from peanut

**DOI:** 10.3389/fpls.2023.1102181

**Published:** 2023-02-09

**Authors:** Yuhui Zhuang, Yasir Sharif, Xiaohong Zeng, Suzheng Chen, Hua Chen, Chunhong Zhuang, Ye Deng, Miaohong Ruan, Shuanglong Chen, Zhuang Weijian

**Affiliations:** ^1^ Center of Legume Plant Genetics and Systems Biology, College of Agriculture, Fujian Agriculture and Forestry University, Fuzhou, China; ^2^ College of Life Sciences, Fujian Agriculture and Forestry University, Fuzhou, China; ^3^ Fujian Seed General Station, Fuzhou, China

**Keywords:** aflatoxins, *Arachis hypogaea*, hepatocellular carcinoma, inducible promoter, pathogens, functional characterization

## Abstract

Peanut is an important oil and food legume crop grown in more than one hundred countries, but the yield and quality are often impaired by different pathogens and diseases, especially aflatoxins jeopardizing human health and causing global concerns. For better management of aflatoxin contamination, we report the cloning and characterization of a novel *A. flavus* inducible promoter of the O-methyltransferase gene (*AhOMT1*) from peanut. The *AhOMT1* gene was identified as the highest inducible gene by *A. flavus* infection through genome-wide microarray analysis and verified by qRT-PCR analysis. *AhOMT1* gene was studied in detail, and its promoter, fussed with the *GUS* gene, was introduced into Arabidopsis to generate homozygous transgenic lines. Expression of *GUS* gene was studied in transgenic plants under the infection of *A. flavus*. The analysis of *AhOMT1* gene characterized by in silico assay, RNAseq, and qRT-PCR revealed minute expression in different organs and tissues with trace or no response to low temperature, drought, hormones, Ca2+, and bacterial stresses, but highly induced by *A. flavus* infection. It contains four exons encoding 297 aa predicted to transfer the methyl group of S-adenosyl-L-methionine (SAM). The promoter contains different cis-elements responsible for its expression characteristics. Functional characterization of *AhOMT1*P in transgenic Arabidopsis plants demonstrated highly inducible behavior only under *A. flavus* infection. The transgenic plants did not show *GUS* expression in any tissue(s) without inoculation of *A. flavus* spores. However, *GUS* activity increased significantly after inoculation of *A. flavus* and maintained a high level of expression after 48 hours of infection. These results provided a novel way for future management of peanut aflatoxins contamination through driving resistance genes in *A. flavus* inducible manner.

## Introduction

Aflatoxin (AF) contamination produced by *Aspergillus flavus* is the key limitation for peanut production worldwide, which not only causes enormous economic losses but also jeopardizes animal and human health. Since the first discovery of aflatoxin-contaminated peanut meals leading to the death of more than 100,000 turkey birds in England in the 1960s (Richard, 2008), aflatoxin contamination has caused heavy loss to human and live stocks now and again (Pandey et al., 2019). Aflatoxins are a class of mycotoxins produced by species of the *Aspergillus* genus, mainly including *A. flavus*, *A. parasiticus*, *A. nomiu*s, and *A. tamarii* (Goto et al., 1996; Payne et al., 2006; Pandey et al., 2019). Among the aflatoxins, B1 and B2 synthesized by *A. flavus* and *A. parasiticus* in peanut and corn are the most toxic secondary metabolites, carcinogenic, hepatotoxic, immunosuppressive, and teratogenic to mankind and animals (Amaike and Keller, 2011; Kensler et al., 2011). As aflatoxin is stable in nature and decomposes at 237–306 °C, it may contaminate ordinary foods like meat, eggs, and milk in the food chain leading to wide detriments to humans (Awasthi et al., 2012; Pandey et al., 2019). *A. flavus* can contaminate peanut during the growth stage, called pre-harvest infection, and after harvest, in drying, storage, transport, and processing, called post-harvest infection (Khan et al., 2020; Soni et al., 2020). The pre-harvest infection in crops is the key to post-harvest contamination (Khan and Zhuang, 2019). How to reduce or even resolve the aflatoxin contamination problem in peanut and peanut products has been attracting global attention.

As an important protein, oil-providing crop, and valuable nutrition source for humankind and animals, peanut is affected by several bacterial and fungal diseases. Peanut seeds develop underground and are in continuous contact with soil fungal microbiota. Aflatoxin-producing fungal species live in soil as conidia or sclerotia, while in plants, they live in mycelial form. Under harsh environmental conditions, sclerotia in the soil develop conidia and probably ascospores (Horn et al., 2009), resulting in an increased population under warm and dry conditions. Under suitable growth conditions, *Aspergillus* species invade peanut seeds and deteriorate peanut yield and quality. Breeding Aflatoxin-resistant varieties are the most effective and safe method for managing aflatoxin contamination. But, so far, no bred or released varieties have shown stable resistance to *A. flavus* in the world (Pandey et al., 2020), which makes aflatoxin control a challenging task.

In recent years, the successes of genetic engineering and breeding methods have made it possible to breed aflatoxin-resistant peanut varieties. Success stories are available in some crops, such as the success of Bt-cotton (Fleming et al., 2018; Gutierrez, 2018), which has highly increased yields and reduced the use of insecticides, and the golden rice producing functional nutritions (Tang et al., 2009). At present, nearly all promoters being used in genetic engineering are constitutive, like CaMV 35S promoter, Ubi, and Act-1 (Kay et al., 1987; McElroy et al., 1990; Cornejo et al., 1993), all of them can direct expression virtually in all tissues without any influence by internal and external factors. These promoters not only waste the energy of plants but also bring some unsafety defects to consumers, which has caused broad transgenic safety concerns. To breed the aflatoxin-resistant peanut varieties, *A. flavus*-induced promoters, which only express when plants undergo *A. flavus* infection, would be clearly a good choice. Inducible promoters are of great importance as they don’t pose an additional load on plant metabolism compared to constitutive promoters (Divya et al., 2019). Thus, identification and investigation of Aflatoxins-induced genes and their promoters should be important and have potential applications in creating resistance peanut to aflatoxin contamination.

Plant O-methyltransferases (OMTs) transfer the methyl group of *S-adenosyl-L-methionine* (SAM) to the hydroxyl group of numerous organic chemical compounds, ultimately resulting in the synthesis of the methyl ether variants of these substances (Struck et al., 2012). Plant OMTs are primarily classified into three groups: C-methyltransferases, N-methyltransferases, and O-methyltransferases (OMTs) (Roje, 2006). O-methyltransferases are involved in concern for cresistance mechanism in crop plants against several fungal and bacterial pathogens. OMT members are reported to have fusarium blight resistance in wheat (Yang et al., 2021). OMT members are involved in lodging resistance in wheat by playing a role in regulating lignin biosynthesis and resistance against various environmental stresses (Nguyen et al., 2016). Yang et al. (2019) reported that a wheat OMT improved drought resistance in transgenic *Arabidopsis* (Yang et al., 2019). Another member of wheat OMT (*TaCOMT-3D*) enhances tolerance against eyespot disease and improves the mechanical strength of the stem (Wang et al., 2018). In maize, OMT members enhance PEG and drought tolerance (Qian et al., 2014). Plant OMTs participate in the structural diversification of different chemical compounds; the methylation alters the solubility and reactivity of natural compounds, resulting in a change in their biological processes (Gang et al., 2002). Due to their important roles in secondary metabolism, OMTs are widely investigated in plants. However, no reports have touched the *OMTs* associated with *A. flavus* infection, especially its highly inducible response to the *A. flavus* inoculation, implying an important use for aflatoxin management.

In searching for *A. flavus*-induced promoters, a genome-wide large-scale microarray study was performed (Chen et al., 2016; Zhang et al., 2017), an O-methyltransferase gene (*AhOMT1*) was found to have a gigantic response to the *A. flavus* infection. Then, we studied the aflatoxin inducible promoter of that O-methyltransferase gene (*AhOMT1*). Our study aimed to identify and clone the *OMT1* and the aflatoxins inducible promoter that can be used to drive aflatoxins-resistant genes for future use. We also performed the functional investigation of this promoter in *Arabidopsis* plants and provided its practical significance. If genes with high resistance under the control of aflatoxin inducible promoters are transformed in peanut, it would be an outstanding achievement in obtaining aflatoxin resistance.

## Materials and methods

### Plant materials and growth conditions

In this study, we used peanut cultivars Minhua-6 (M6), Xinhuixiaoli (XHXL), and *Arabidopsis* Columbia-0 (Col-0). XHXL is a highly *A. flavus*-resistant variety. The peanut and *Arabidopsis* seeds were maintained by the Oil Crops Research Institute, Fujian Agriculture and Forestry University. Peanut plants were grown in research fields and the greenhouse of Fujian Agriculture University. *Arabidopsis* plants were grown in small plastic pots (8 cm diameter). Peanut and *Arabidopsis* plants were maintained at 25°C and 16/8 h day/night photoperiod.

### Fungal strain growth and inoculation

A highly toxic strain of *A. flavus* (AF2) was grown on Potato Dextrose Agar medium PDA (Potato=200g, Dextrose=20g, Agar=15g, water up to 1L). The fungal isolates were grown on PDA in a 100 mL flask at 28 °C for one week. Green spores of AF2 were collected in a solution of 0.01% tween 20. The spore concentration was diluted to 1.9 ×10^6^ conidia mm^-1^ (Li et al., 2006). The spore solution was used to spray the peanut leaves, and samples were taken at 3h, 6h, 9h, 12h, 24h, 36h, and 48h after inoculation. Non-treated sampled (0h) were used as a control to compare the expression of the *AhOMT1* gene.

### Selection of aflatoxins inducible gene

The screening of aflatoxin inducible genes was based on our previous microarray studies (Chen et al., 2016; Zhang et al., 2017). Differential expression profiles were compared among different peanut tissues, including the maturing pods which were infected by *A. flavus* plus drought (PAFDR, peanut *A. flavus* inoculation plus drought), dealing with drought (PDR-af, peanut in drought stress, no *A. flavus* inoculation) and normal watering (PAFCK, peanut without *A. flavus* inoculation as for control), using microarray with oligonucleotides of 60bp containing 12x135K arrays. Trizol reagent was used to extract the total RNA, and array hybridization, washing, scanning, and data analysis were performed according to NimbleGen’s Expression user’s guide for expression comparisons. From the microarray expression, *A. flavus* inducible specific expression gene fragments were isolated. A member of the peanut OMT family, “Isoliquiritigenin 2’-O-methyltransferase”, was found as a highly upregulated gene.

### Cloning and analysis of the *AhOMT1* gene

The full-length *AhOMT1* gene was cloned from the peanut pericarp cDNA infected by *A. flavus*. Based on the target gene sequence, the full-length gene was amplified by RACE (Rapid Amplification of cDNA Ends) with the special primers, RACE-F and *AhOMT1*-R for the 5’-upstream fragment cloning; RACE-R and *AhOMT1*-F for the 3’ downstream fragment cloning. Primer sequences are given in **Table S2**. The *“Isoliquiritigenin 2’-O-methyltransferase”* gene with an mRNA Id “AH13G54850.1” is present on the chromosome 13^th^ of cultivated peanut (http://peanutgr.fafu.edu.cn/) (Zhuang et al., 2019). As this gene is being studied for the first time in peanut, we renamed it *AhOMT1* for convenience.

We used BioXM 2.6 software to predict the open reading frame of the splicing sequence. Sequence homology analysis was performed using BLASTP at NCBI, TAIR (https://www.arabidopsis.org/) (Lamesch et al., 2012), and Legume Information System LIS (https://legacy.legumeinfo.org/) (Gonzales et al., 2005). The protein sequence was also submitted to ScanProsite and NCBI databases to analyze the protein binding sites and functional domains. Protein physical and chemical parameters were analyzed by ExPASy (Gasteiger et al., 2003). The evolutionary reconstructions of OMT1 proteins from different legume species and peanut were studied by constructing a rooted phylogenetic tree. Protein sequences were aligned by Clustal Omega (https://www.ebi.ac.uk/Tools/msa/clustalo/), and a rooted tree was constructed by Dendroscope 3 (Huson and Scornavacca, 2012).

### Cloning and analysis of *AhOMT1* promoter

Cloning of the *AhOMT1* promoter is based on the previous Nested PCR analysis. Genomic DNA was extracted from the young roots of peanut M6 using the CTAB method. Peanut Genome Walker libraries were constructed using our own library’s approach. The genomic DNA was incompletely digested by three restriction enzymes (AseI, EcoRI, and HindIII) and then ligated the fragments to adapters to construct three genomic libraries using the adaptor primers, ongAd, ShortHindIII, ShortEcoRI, and ShortAseI (**Table S2**).

Genomic libraries were used as the PCR temple to obtain the upstream sequence by Flanking PCR. Two forward primers (AP1, AP2-C) and two reverse primers (AhOMT1-SP1, AhOMT1-SP2) were designed to clone the promoter of the *AhOMT1* gene (**Table S2**). Finally, a primers pair was designed to clone the promoter from the genomic DNA of peanut; the forward primer (AhAF7-F) was designed upstream of the coding sequence, and the reverse primer (AhAF7-R) was designed at the *AhOMT1* gene sequence (**Table S2**). The PCR amplified fragment was visualized through 2% agarose gel, purified by TIANGEN Universal DNA Purification Kit (TIANGEN Biotech Beijing, China), and sequence was verified by Beijing Genomics Institute (BGI, Shenzhen, China).

A 1698 bp region upstream of the start codon of the *AhOMT1* gene was selected for promoter analysis. *Cis-*regulatory elements of the promoter region were predicted by the PLACE database (https://www.dna.affrc.go.jp/PLACE/?action=newplace) (Higo et al., 1998) and the PlantCARE database (http://bioinformatics.psb.ugent.be/webtools/plantcare/html/) (Rombauts et al., 1999).

### Verification of *A. flavus* inducible expression of *AhOMT1* gene

Following the *A. flavus* inoculation of peanut leaves, samples were taken and preserved at -80°C. RNA was extracted by the Cetyl Trimethyl Ammonium Bromide (CTAB) method with few modifications (Chen et al., 2019). Following the manufacturer’s protocol, 1µg RNA was reverse transcribed to synthesize the cDNA with the PrimeScript 1st strand cDNA Synthesis Kit (Takara, Dalian, China). To check the real-time expression of the *AhAOMT1* gene in response to *A. flavus* infection, a qRT-PCR reaction was performed using the MonAmp™ ChemoHS qPCR Mix (Monad Biotech, Wuhan, China). The reaction was prepared according to the product guidelines. The peanut *Actin* gene was used as a control (Chi et al., 2012), and the reaction was carried out in Applied Biosystems 7500 real-time PCR system (USA) at 94°C (1 min), 60°C (1 min), and 72°C (1 min) for 40 cycles. Primers used for RT-qPCR are given in **Table S2**.

### Vector construction and transformation into *Arabidopsis*

A two-step Gateway cloning system was used to construct the plant expression vector using the pMDC164 vector. The pMDC164 vector is a plant expression vector that contains the *GUS* reporter gene and hygromycin antibiotic resistance gene. After sequence verification, the *AhOMT1* promoter fragment was ligated between *attP* sites of entry vector pDONR-207 by BP reaction. The promoter sequence was verified again and then cloned into the pMDC164 vector between *attR* sites by Gateway LR cloning with the primers containing universal Gateway adapter sequences.

Constructed vector *AhOMT1P::GUS* was transformed into *A. tumefaciens* (GV3101) and KM+ (50 µg/mL) resistance colonies were selected on YEB and verified by PCR. The Transformed *A. tumefaciens* cells were grown to the OD_600_ of 1.0-1.5. Bacterial cells were harvested by centrifugation and resuspended sucrose solution (5%), also containing Silwet L-77 (0.02%) and Acetosyringone (AS) (100µg/mL). The floral dip method (Clough and Bent, 1998) was employed for the genetic transformation of mature *Arabidopsis* plants. Opened flowers were clipped off before floral dipping, and the transformation was repeated after five days. Plants were grown normally following the transformation procedure, and mature seeds were collected. T0 seeds were sterilized with ethanol (75%) and 10% H_2_O_2_ and grown on MS medium containing 50µg/mL Hygromycin for screening of positively transformed plants. From Hygromycin-resistant plants, eight were randomly selected for genetic verification for the presence of promoter by PCR with the promoter and *GUS* gene-specific forward and reverse primers (**Table S2**). PCR-verified plants were grown to get the homozygous T3 generation for further functional studies. In each generation, greater care was carried out to avoid outcrossing, while Hygromycin and PCR-based verification were repeated in each generation.

### Functional study of *AhOMT1* gene in response to *A. flavus*

The *AhOMT1* promoter expression was analyzed by studying the behavior of the *GUS* gene in transgenic plants. Histochemical staining and quantitative expression of the *GUS* gene were checked in response to *A. flavus* infection. Transgenic plants were divided into three groups, each infected with *A. flavus*. For *A. flavus* infection, the spore solution was prepared as mentioned above and used to spray the young plants. Leaf samples were collected before treatment (0h) and after infection, 1h, 3h, 6h, 12h, 24h, and 36h. GUS staining solution 2mM 5-Bromo-4-chloro-3-indolyl β-D-glucuronide (X-Gluc) was prepared in 10 mM EDTA, 0.1% Triton X-100, 2 mM Potassium ferricyanide, 50 mM sodium phosphate buffer, and 2 mM Potassium ferrocyanide (Jefferson et al., 1987). Samples were incubated in GUS solution at 37°C for 12 hours. After that, samples were washed and decolorized with 75% ethanol. Photographs of stained samples were taken with an OLYMPUS microscope (BX3-CBH) and digital camera. For quantitative expression analysis of *GUS* gene, qRT-PCR was performed with samples taken from *A. flavus*-stressed plants. RNA was extracted with Trizol reagent, as mentioned above. Transgenic plants were also treated with different plant hormones, including salicylic acid (SA, 3mmol/L), ethephon (ETH, 1mg/ml), Brassinolide (BR, 0.1 mg/L), abscisic acid (ABA, 10μg/ml) and paclobutrazol (PAC, 150mg/L) to check that either *AhOMT1* promoter is affected by these hormones or not. Plants were treated with distilled water as a control group.

## Results

### Selection and verification of *A. flavus* inducible *AhOMT1* gene

Aflatoxin inducible genes were selected from a previous microarray expression study. Microarray analysis was performed on peanut cultivar Minhua-6 plants treated with drought, a combination of drought and *A. flavus* infection, and a control group. Plants with normal watering were taken as control group. Gene with high microarray expression under drought and *A. flavus* infection were selected as *A. flavus* inducible genes. Among the upregulated genes, a member of peanut O-methyltransferase family “Isoliquiritigenin 2’-O-methyltransferase” (*AhOMT1*) with the probe Id “GFBZZHF01DILSM” was found to be highly inducible. Expression levels of ten highly *A. flavus* inducible genes are shown in **Figures S1A, B** (Log2 normalized microarray expression values are used), while the relative expression values are given in **Table 1**. High-density oligo-nucleotide microarray with 100,000 unigenes, including the *AhOMT1* gene and relative expression levels of the *AhOMT1* gene across different tissues, is given in **Figures S1A–C**. Expression level of the *AhOMT1* gene under control and drought conditions was almost similar, but it recorded a high increase in microarray expression in response to a combination of drought and *A. flavus* infection. An almost 200-fold increase was observed upon *A. flavus* inoculation. *AhOMT1* recorded the highest increase in expression among inducible genes, so it was selected for the promoter analysis.

**Table 1:** Microarray expression of some *A. flavus* inducible genes.

Probe Id		Microarray expression			
	Gene name	Normal watering	Drought	Drought + *A. flavus*	Folds of upregulation
GFBZZHF02GJYBV	40S ribosomal protein S5 (Fragment)	12	14	9194	656.71
GALJ6PP01B5DBY	Probable F-box protein At4g22030	15	13	3562	274.00
GAYHEI101EGW7N	Protein GLUTAMINE DUMPER 1	888	31	7672	247.48
GALJ6PP01DP2GR	EG45-like domain containing protein	13	11	2751	250.09
GD3IWDR02CZXC6	Uncharacterized protein	19	22	4752	216.00
GFBZZHF01DILSM	Isoliquiritigenin 2’-O-methyltransferase	49	86	17355	201.80
GD3IWDR02DXEVJ	Laccase-14	31	29	4462	153.86
GFBZZHF02F852U	Probable inactive purple acid phosphatase 1	11	26	3856	148.31
GALJ6PP01A9Y8O	Unknown function	17	26	3793	145.88
GAYHEI101CFH5I	Cysteine-rich receptor-like protein kinase 3	40	28	2358	84.21

* Microarray expression under different treatments.

**Figure 1:**
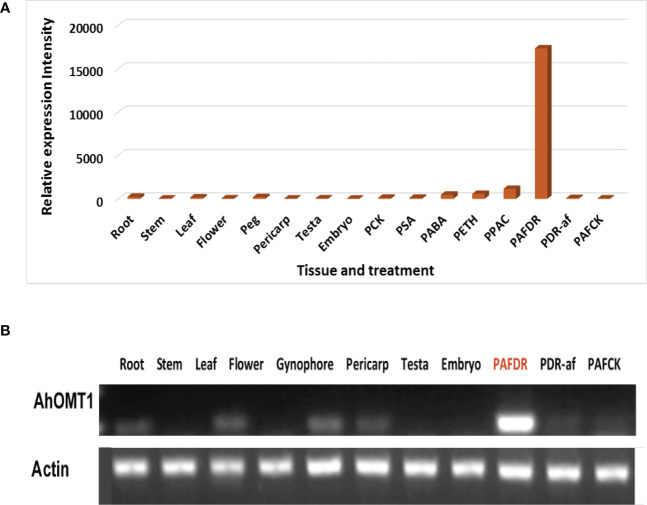
*A. flavus* inducible gene screening and *AhOMT1* expression in peanut. **(A, B)** *AhOMT1* gene showed very week or no expression in different tissues (root, stem, leaf, flower, pericarp, testa, and embryo), but gigantic upregulation induced by *A. flavus* by both microarray and RT-PCR analysis. The DNA template from the pericarp treated with drought (PDR-af), drought plus *Aspergillus flavus* infection (PAFDR), and normal watering (PAFCK).

For evaluation of *AhOMT1*, RT–PCR was performed using total RNA obtained from root, stem, leaf, flower, pericarp, testa embryo, and aflatoxins-inoculated pericarp tissues. The result showed that the gene was nearly unexpressed in these peanut organs without any stress treatments (**Figures 1A, B**). We also performed the semi-quantitative RT-PCR analysis and got consistent results. However, *AhOMT1* detected high expression in the peanut pericarp infected with *A. flavus* (**Figure 1B**). These findings are in accordance with the analysis of the cDNA microarray in two sets of *in silic* expression for *AhOMT1* gene evaluation (**Figure S1C**; **Table S1-1, 2**). The result confirmed that *AhOMT1* is an *A. flavus*-induced gene.

**Figure 2:**
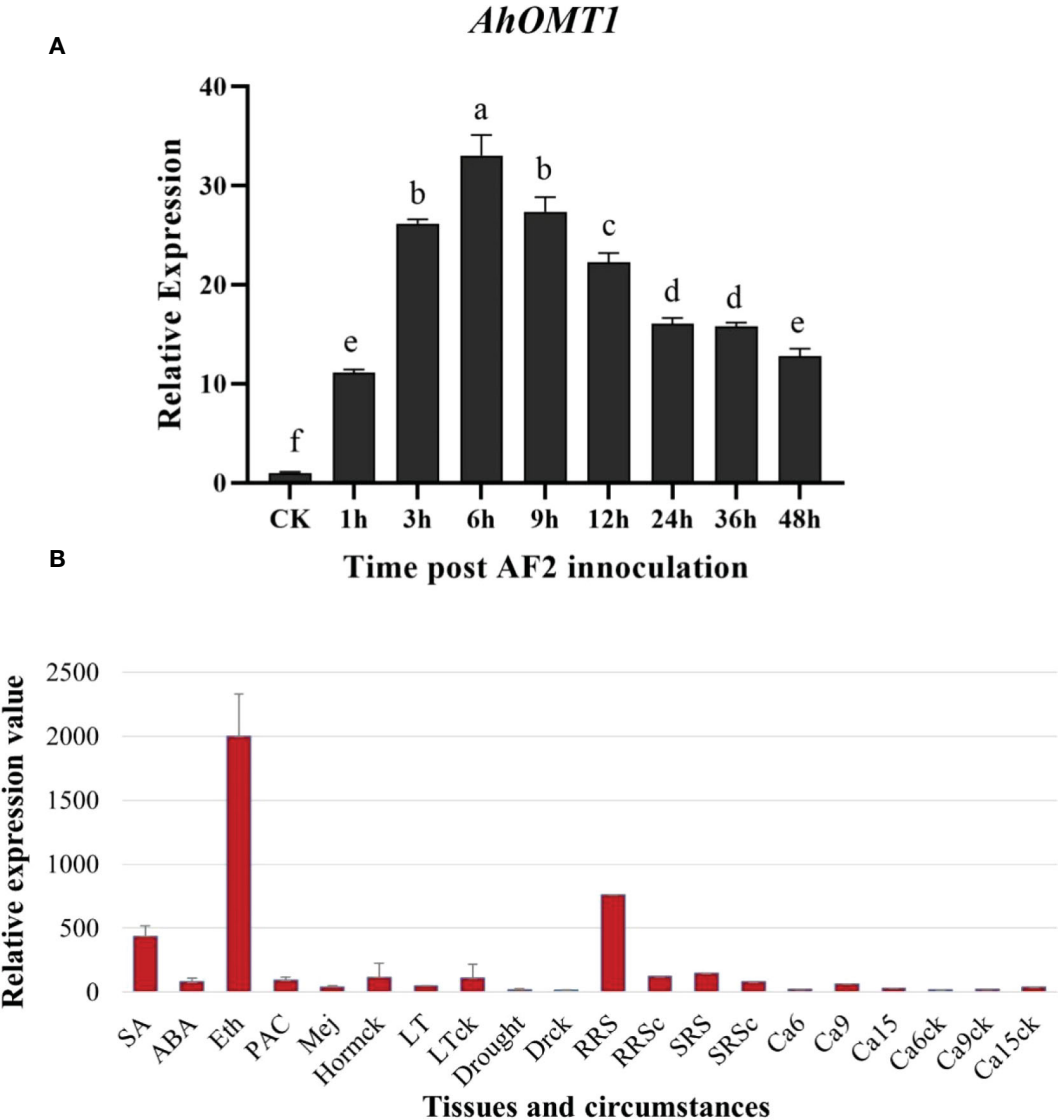
Characteristics of *AhOMT1* expression in peanut. **(A)** Expression analysis of *AhOMT1* gene in response to *A. flavus* infection determined by qRT-PCR. *AhOMT1* showed high level of expression upregulation upon *A. flavus* infection in peanut leaves. Expression was increased after one hour of inoculation that showed increasing trend upto 6 hours. After 6 hours, expression showed a decreased trend but still was very high as compared to non-treated leaves. **(B)** *AhOMT1* gene demonstrated little responses to hormones and environmental stresses besides Ethephon treatment (Eth) with upregulation. SA, salicylic acid; ABA, abscisic acid; PAC, paclobutrazol; Mej, Methyl jasmonate; LT, low temperature; RRS, *Ralstonia solanacearum* inoculation on resistance peanut; RRSc, resistance cultivar without inoculation; SRS, *R. solanacearum* inoculated on susceptible cultivar. SRSc, susceptible cultivar without inoculation; Ca6/9/15: embryo in deficient calcium at 6, 9 and 15 days after pegging; Ca6/9/15ck: embryos in sufficient calcium at 6, 9 and 15 days after pegging.

Further evaluation of the *AhOMT1* inducible behaviors was performed by qRT-PCR analysis and another set of microarray analyses with various stresses (**Figures 2A, B**, **S1-B**). For the qRT assay, the peanut plants were challenged with *A. flavus*. The expression level of *AhOMT1* in leaves was observed at 1h, 3h, 6h, 9h, 12h, 24h, 36h, and 48h after inoculation. Data were analyzed by the 2^-ΔΔCt^ method while using Actin as control gene. *AhOMT1* gene was expressed gradually upon *A. flavus* treatment at 1h, 3h, and 6h after inoculation (**Figure 2A**). After 6h, *AhOMT1* showed decreased expression, but the expression level was still much higher compared to the control (without treatment). Microarray studies showed that compared with actin-7, *AhOMT1* expressed weakly in different organs and tissues and did not respond to environmental challenges such as low temperature, drought, deficient Ca^2+^, bacterium *Ralstonia solanacearum* inoculation, and also to hormones treatments but upregulated by Ethephon (**Figure S2B**; **Tables S1, 2**). These results indicate that the *AhOMT1* gene is highly expressed under *A. flavus* infection.

### Cloning and characterization of *AhOMT1* gene

**Figure 3:**
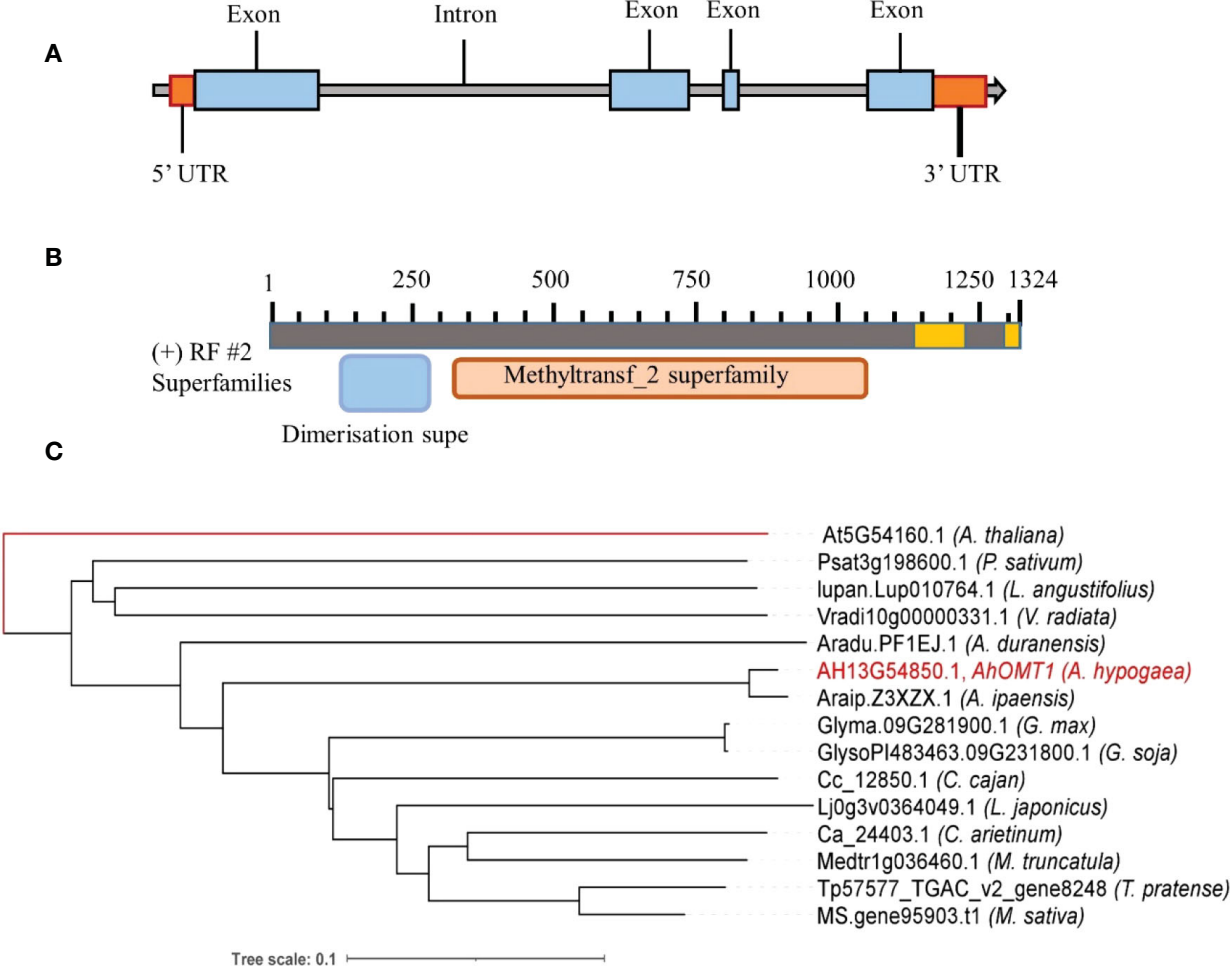
Structure and classification of *AhOMT1* gene. **(A)** Structure of *AhOMT1* gene. **(B)** Functional domains structure of *AhOMT1*. **(C)** Phylogenetic relationship of *AhOMT1* gene with *OMT1* genes of other legume species. Unrooted tree was constructed by taking *AtOMT1* as reference.

The cDNA of the *AhOMT1* gene was first obtained from *Arachis hypogaea* cv. Minhua-6 pericarp cDNA infected with *A. flavus* by RACE approach using the designed specific primers (**Table S2**). After obtaining the 5’ upstream and 3’ downstream cDNA sequence of *AhOMT1*, two specific oligo primers were designed to clone the entire cDNA and genomic DNA. The gene *AhOMT1* contained a 1,293 bp full-length cDNA (not counting 31bp of polyA) comprised of a 241 bp 5’UTR, an 894 bp long ORF and a 158bp of 3’UTR (**Figure S2**). The translated coding sequence encodes a 297 amino acid protein. The comparison of genomic and cDNA sequences showed that ORF contains three introns (**Figures 3A**, **S2**) of 130 to 816 bp length, and all introns had a usual 5’GT and 3’AG ends (Ballance, 1986). This supported but revised the annotation of *AhOMT1* gene structure with 1086 bp ORF and 49 bp 5’UTR (*AH13G54850.1*) in Chromosome 13, a 192bp upstream fragment being predicted to be a coding region (Zhuang et al., 2019). Presence of Methyltransf_2 superfamily domain provided that *AhOMT1* belongs to O-Methyltansferase gene family (**Figure 3B**).

*OMT1* homologous genes from other legume species were identified from LIS and TAIR databases. The percent identity was found by BLASTP in DNAMAN8.0 software. As *AhOMT1* is present on 13^th^ chromosome (B sub-genome), derived from ancestral species *A. ipaensis*, and the results were consistent to expectation. It showed 97.41% identity with *A. ipaensis* (Araip.Z3XZX.1), while identity with *A. duranensis* (Aradu.PF1EJ.1) is 56.15%, indicating a rapid diversification of it. *AhOMT1* showed 70.99% identity with *G. max* OMT1 homolog (Glyma.09G281900.1) and 57.76% identity with *Arabidopsis* (At5G54160.1) (**Table 2**). Phylogenetic analysis revealed a well-supported division between methyltransferase family groups, and the *AhOMT1* lies within a distinct clade containing the *AhOMT1* (**Figure 3C**), providing further support that *AhOMT1* is a gene encoding Isoliquiritigenin 2’-O-methyltransferase.

**Table 2:** Comparison of *AhOMT1* to homologous genes from other legume species.

Species	Genes description	Accession number	Identity %
*Glycine max*	O-methyltransferase	Glyma.09G281900.1	70.99
*Medicago truncatula*	Caffeate O-methyltransferase	Medtr1g036460.1	61.58
*Lotus japonicus*	O-methyltransferase 1	Lj0g3v0364049.1	61.45
*Arabidopsis thaliana*	O-methyltransferase 1	At5G54160.1	57.76
*Arachis ipaensis*	O-methyltransferase 1	Araip.Z3XZX.1	97.41
*Arachis duranensis*	O-methyltransferase 1	Aradu.PF1EJ.1	56.15
*Arachis hypogaea*	Isoliquiritigenin 2’-O-methyltransferase	AH13G54850.1	100
*Arachis hypogaea*	Isoliquiritigenin 2’-O-methyltransferase	AH10G32250.1	98.6

The gene accession numbers are as given in LIS, TAIR, and PGR databases.

The protein sequence analysis by ExPASy showed that the *AhOMT1* had a molecular weight (MW) of 33532.5 and a theoretical isoelectric point (pI) of 6.49. The instability index (II) is computed to be 44.34, indicating an unstable protein. The protein was predicted to be a hydrophilic protein with no signal peptide region (**Figure S3A–4B**). And the prediction of protein hydropathicity also showed that *AhOMT1* is a hydrophilic protein (**Figure S3C**). These indicated that *AhOMT1* might be a peripheral protein. The conserved domain analyzed by ScanProsite showed that the domain contains five S-adenosyl-L-methionine binding sites and a proton acceptor site (**Figure S3D**). The protein binding sites and functional domains of the *AhOMT1* gene analyzed by the NCBI database suggested that the gene is a member of the Methyltransf_2 superfamily (**Figure S3E**). This family contains a range of O-methyltransferases. These enzymes utilize S-adenosyl methionine (Kozbial and Mushegian, 2005). All these indicated this gene encoded a putative O-methyltransferase.

### Isolation of *AhOMT1* promoter sequences

Genomic DNA was extracted from leaf samples of peanut (**Figure S4A**). 4µg genomic DNA was incompletely digested with the restriction enzyme *AseI, EcoRI*, and *HindIII* for 5min, 15min, and 25min, respectively (**Figure S4B**). Then we ligated the corresponding Adapter to the digestion products of the three restriction enzymes by T4 DNA Ligase overnight at 20 °C.

With the digested DNA connected with the complementary adaptors as the template, more than 1700bp fragment was isolated by three times nest PCR. The promoter was amplified using genomic DNA (50ng/μl) with primers AhAF7-F and AhAF7-R. After agarose gel electrophoresis, we got two bands that showed in **Figure S4C**. We linked them to the vector pMD18-T and sequenced them. The sequencing results showed both bands containing a 197bp downstream fragment that overlapped the upstream *AhOMT1* gene with the same sequence, indicating that the cloned promoters were correct starting from *AhOMT1*. Detach the overlapped sequence with the *AhOMT1* gene, the length of the two promoters region of the *AhOMT1* before the ATG were 1,690bp and 1103bp, respectively (**Figure S5**). We named them *AhOMT1* promoter L and *AhOMT1* promoter S. The two sequences, long and short, were then approved to be the promoters of *AhOMTs* from respective Ah13G54850.1 and Ah10G32250.1 genes (Zhuang et al., 2019). For future studies, we used the AhOMT1 L promoter from high aflatoxin resistance peanut cultivar XHXL.

### Analysis of *cis*-regulatory elements of *AhOMT1* promoter

**Figure 4:**
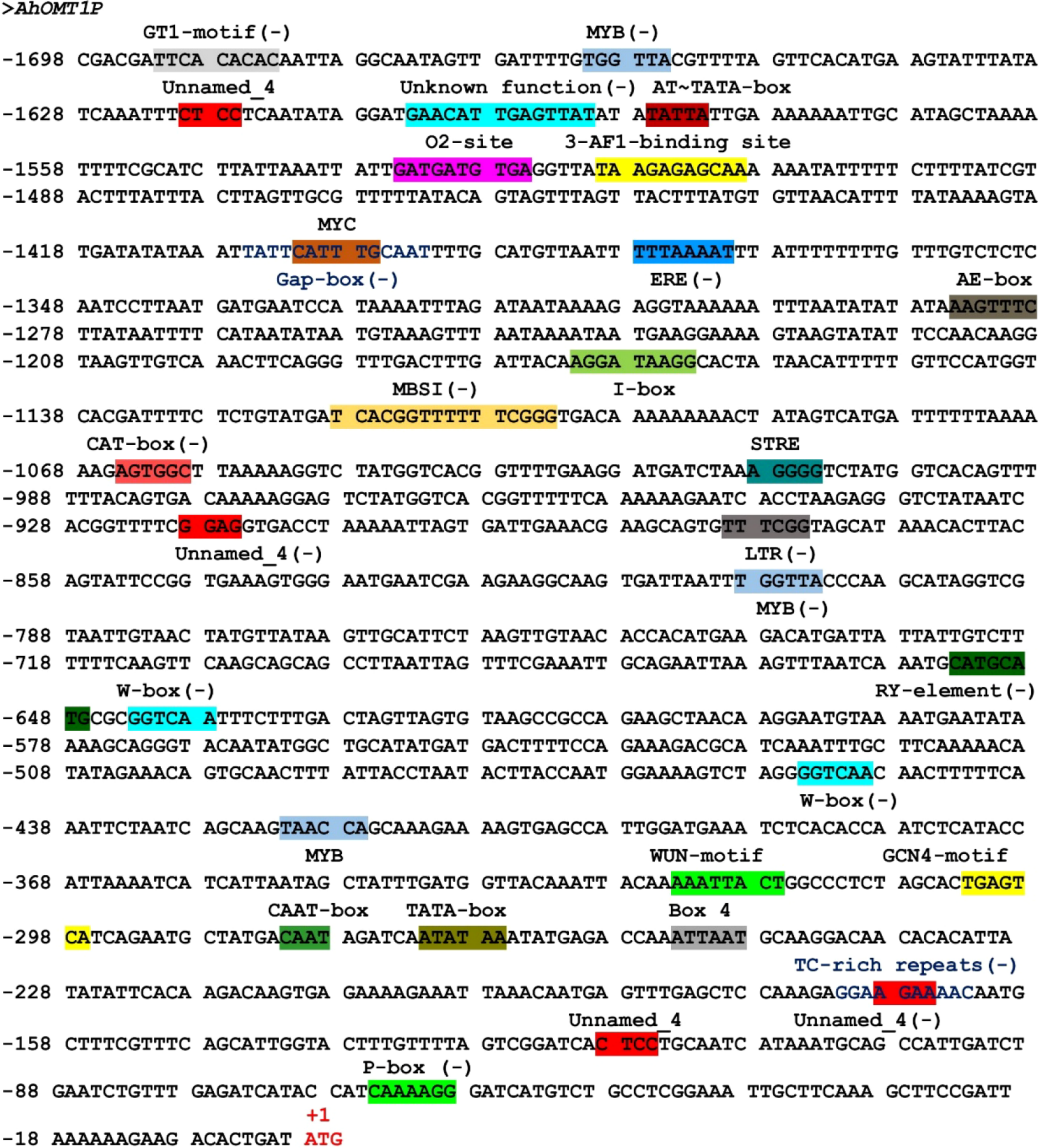
Sequence analysis of *AhOMT1* promoter. Presence of *cis*-elements in promoter sequences predicted by the PlantCARE database.

The *cis-acting* elements of the L promoter region of the *AhOMT1* gene were predicted by online databases viz, PLACE (Higo et al., 1998) and PlantCARE (Rombauts et al., 1999). A 1698 bp sequence upstream of the *AhOMT1* coding region contained the core elements, including the TATA box, required for precise initiation of transcription (Grace et al., 2004), the CAAT box needed for tissue-specificity (Shirsat et al., 1989). Other key elements include light-responsive elements; Box 4, GATA-motif, GT1-motif, Gap-box, and I-box. Several others were also present, including defense-related elements, WUN-motif, MYB binding sites, ethylene-responsive element ERE, and wound-responsive elements. Zein metabolism-responsive element (O2-site), flavonoid biosynthesis elements (MBSI), and seed-specific elements (RY-element) were also present. More details of *cis*-regulatory elements in *AhOMT1P* are given in **Figure 4**. The presence of these elements suggests that *AhOMT1P* can be a suitable candidate for a gene’s own promoter. Some novel elements were predicted in promoter region, except these previously identified *cis*-elements (**Figure 4**). Further details for the *cis*-regulatory elements predicted in *AhOMT1* promoter through PlantCARE database are given in **Table S3**. The *AhOMT1* promoter sequence are given in **Supplementary File 1**, while the elements predicted through the new PLACE database are given in **Table S4**.

### Generation of transgenic plants with *AhOMT1P: GUS* fission unit

**Figure 5:**
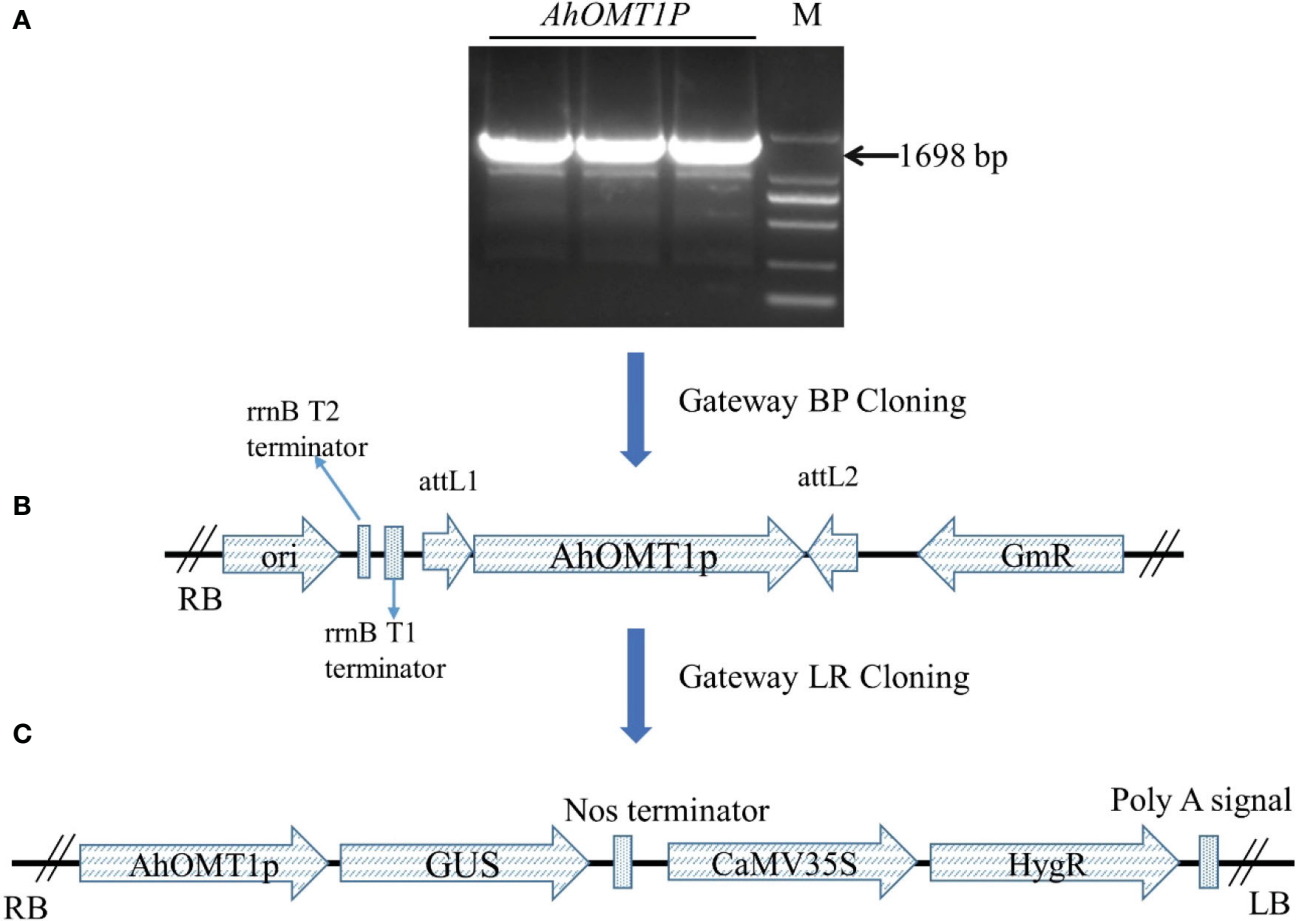
Construction of vectors using the backbone of *pMDC164* vector by Gateway cloning. **(A)** Amplification of *AhOMT1* promoter, M=2kb marker. **(B)** Construction of Gateway entry clone using *pDONR207* vector. **(C)** Construction of Gateway expression vector using the backbone of binary vector *pMDC164*.

**Figure 6:**
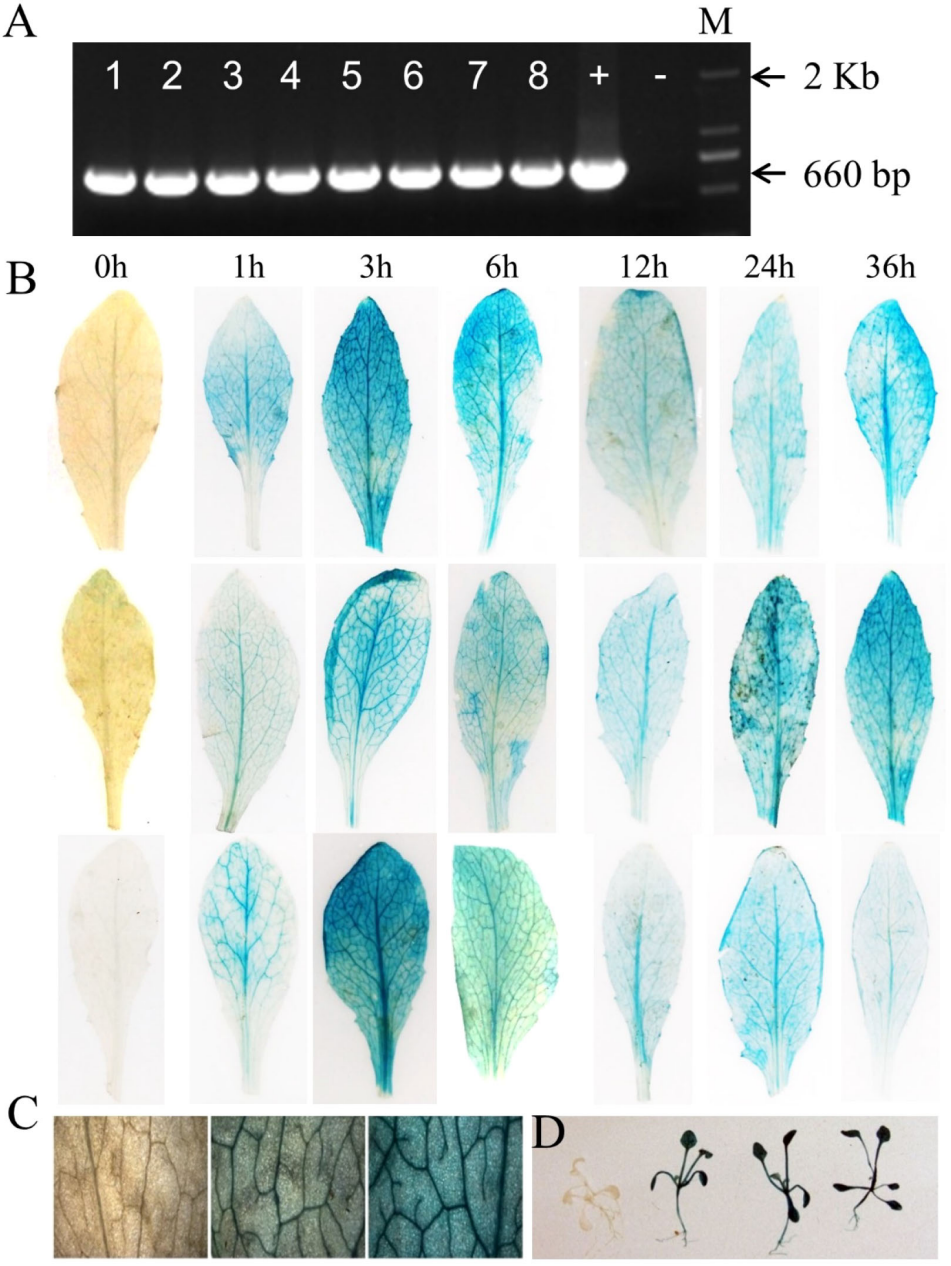
Evaluation of the function of *AhOMT1* promoter. **(A)** Confirmation of transgenic *Arabidopsis* plants with *AhOMT1P* (660bp fragment) PCR amplification with promoter-specific forward and *GUS* gene specific reverse primers pair. *Arabidopsis* Col-0 was used as negative control, and Gateway LR constructs used as positive control. M shows 2kb marker. **(B)** Strong GUS staining of *AhOMT1P* transgenic leaves is only induced by *A. flavus* infection with spores after 1 h of infection. *GUS gene* was not active in non-treated leaves. GUS staining was present in leaf samples at all time points after inoculation. **(C)** Magnified leaf surface staining indicated that *AhOMT1P* activated the *GUS* gene expression after infection with *A. flavus* spores. **(D)** Transgenic seedlings showing deep GUS staining only after *A. flavus* challenging.

A 1698bp upstream region of the *AhOMT1* gene was PCR amplified (**Figure 5A**) using the DNA of a high *A. flavus*-resistant peanut variety XHXL by promoter-specific primers (**Table S2**). Following the Gateway BP and LR cloning steps, the expression vector *AhOMT1P::GUS* was successfully constructed (**Figures 5B, C**) and transformed to *A. tumefaciens*. Genetic transformation of *Arabidopsis* plants was carried out by the floral dip method, and hygromycin-resistant positive transgenic plants were confirmed by PCR amplification. Eight plants showing resistance to Hygromycin were selected for PCR confirmation (**Figure 6A**).

### Characterization of *AhOMT1P* expression function

Histochemical GUS staining was checked under control (ddH_2_O) and *A. flavus* infection. Transgenic *Arabidopsis* plants were sprayed with *A. flavus* spores. Leaf samples were taken before *A. flavus* inoculation and 1h, 3h, 6h, 12h, 24h, and 36h after spray and incubated in staining solution to check the histochemical expression of the *GUS* gene. Results showed that staining was not present in control plants (0h, before spray). At the same time, a strong blue color was present in treated leaves at all time points, indicating that the *GUS* gene was induced in response to *A. flavus* treatment after 1h, and it continuously showed high expression at 3h, 6h. 12h, 24h, and 36h samples with three replications (**Figure 6B**). More results for GUS staining of whole leaves of transgenic *Arabidopsis* plants can be seen in **Figure S6**. While the small sections of stained leaves are shown in **Figures S7**, **6C**. Small seedlings of transgenic plants are shown in **Figures S8**, **6D**. These results showed that the *AhOMT1* promoter was inactive under normal conditions, as staining was not present in non-treated leaves. *AhOMT1P* was induced only after *A. flavus* infection. These results supported that the *AhOMT1* gene is an *A. flavus* inducible gene and a suitable candidate for the native promoter of a gene. Based on these results, it can be predicted that the *AhOMT1* promoter can activate anti-aflatoxin genes upon *A. flavus* infection. Using this promoter to drive aflatoxin resistance genes in transgenic peanut will provide an excellent way to improve the aflatoxin resistance of transgenic peanuts.

### Quantitative expression of *AhOMT1P* controlled *GUS* gene in response to *A. flavus* infection and plant hormones

**Figure 7:**
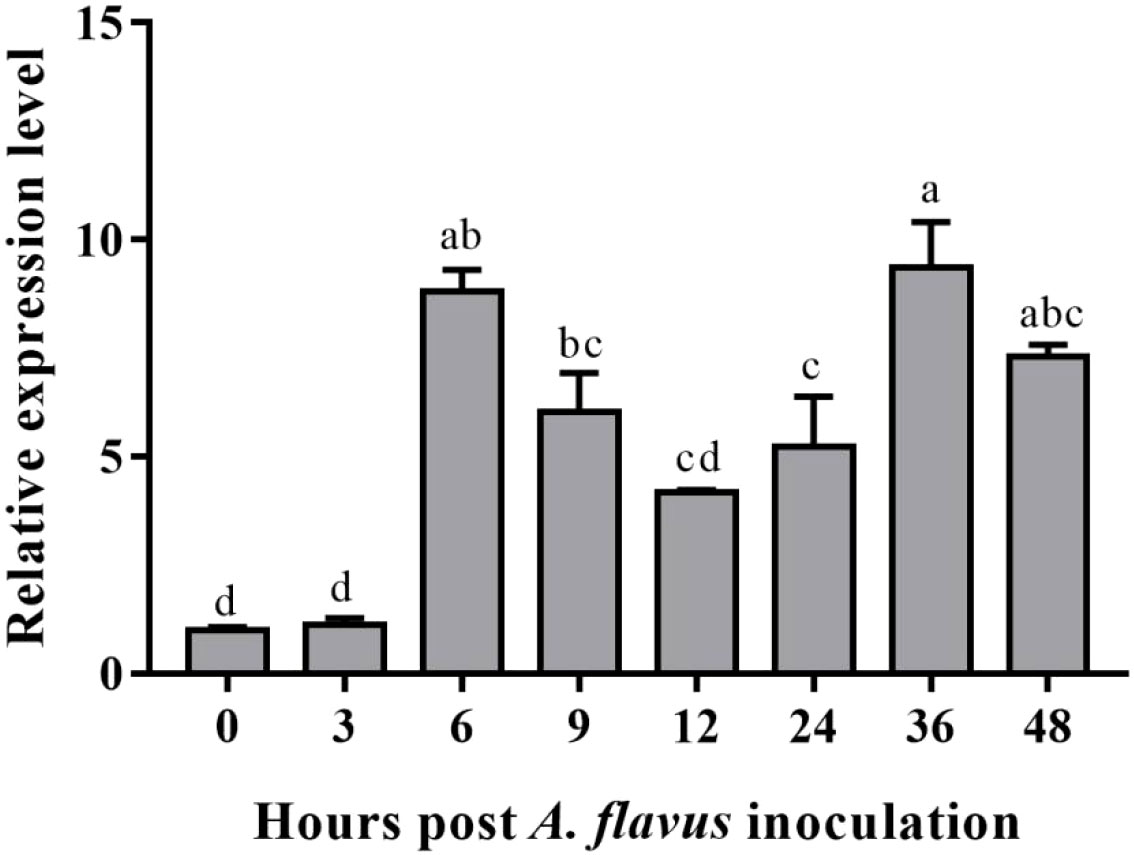
Quantitative expression of *GUS* gene in response to *A. flavus* infection. The *GUS* gene recorded an increased expression at 6h post inoculation and maintained higher expression even after 48 hours. The qRT-PCR data were analyzed by ΔΔCT method, statistical significance was assessed by LSD model with *α=0.05*.

**Figure 8:**
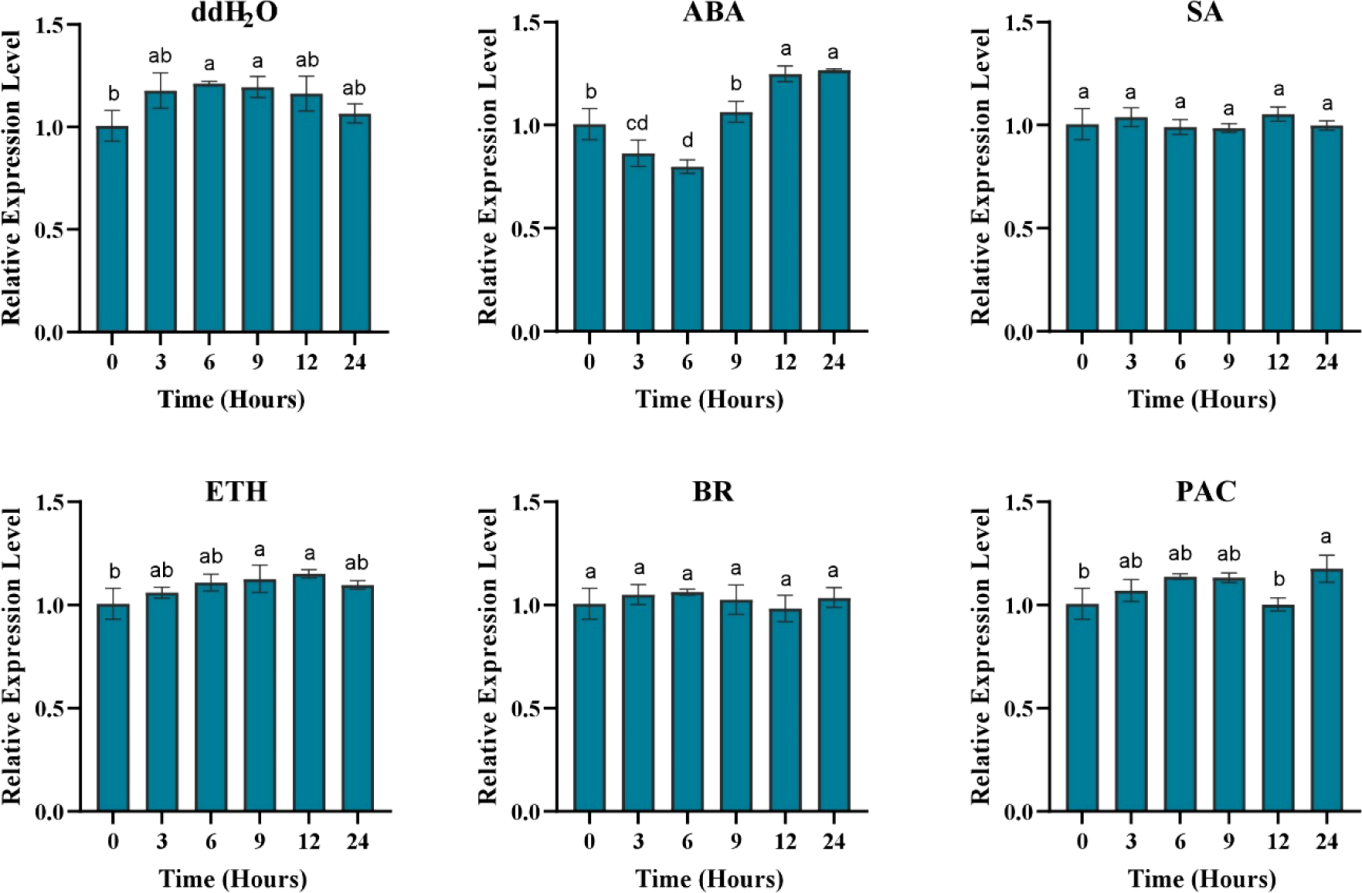
Quantitative expression of *GUS* gene in response to different hormone treatments. The *GUS* gene showed reduced expression in response to different hormones and ddH_2_O spray at all time points. ABA, abscisic acid; BR, Brassinolide; ETH, ethephon; PAC, paclobutrazol; and SA, salicylic acid. Data were analyzed by analysis of variance (ANOVA, *α=0.05*).

Because the GUS staining just reflects continuous gene expression in *A. flavus* treated leaves after 1h, to quantify gene expression intensity at different time points for comparison more detailedly after *A. flavus* infection, the *GUS* gene expression was determined by qRT-PCR. The expression of the *GUS* gene was enhanced in response to *A. flavus* infection after 6 hours, and this increased expression was still maintained after 48 hours of inoculation (**Figure 7**). Although the *GUS* gene showed a reduced expression after six hours, the expression level was still too high compared to non-treated plants. Maximum expression was observed at 36 hours post-inoculation. The expression level of *AhOMT1* promoters under different phytohormones stress was determined to check that either this promoter is only induced by *A. flavus* or it can be induced by different hormones. So, the real-time expression of the *GUS* gene in transgenes treated with hormones was determined by qRT-PCR. For that purpose, transgenic *Arabidopsis* plants were treated with abscisic acid, Brassinolide, ethephon, paclobutrazol, and salicylic acid solutions. Under all hormonal treatments and distilled water, the *GUS* gene did not show any remarkable increase in expression. The expression level of the *GUS* gene was a little reduced in some cases and a little increased in others. Overall, the expression pattern was comparable to the transcriptome expression of the *AhOMT1* gene in peanut compared to control or non-treated plants (**Figure 8**). The *GUS* gene’s expression profiling in response to different hormonal treatments provided that the *AhOMT1* promoter did not induce the expression of the *GUS* gene under hormone treatments. Deep GUS staining and increased quantitative expression of the *GUS* gene upon *A. flavus* infection is indicative of the inducible behavior of the *AhOMT1* promoter. Our results are supported with our transcriptome analysis in another peanut cultivar (Zhuang et al., 2019) (**Table S1**). The same results were obtained that *AhOMT1P* was non-responsive to any hormone treatment, and very weak expression was found in all tissues or organs in peanut. Thereby, it is evident that plant hormones do not induce the *AhOMT1* promoter. These results collectively suggest that *AhOMT1P* is a suitable candidate for *A. flavus* resistance transgenic breeding.

## Discussion

As an important geocarpic crop supplying humans with oil, food, and feed worldwide, peanut is vulnerable to many pathogens and diseases (Severns et al., 2003; Pandey et al., 2019; Soni et al., 2020). Aflatoxin is known as the strongest carcinogen caused by species of the genus *Aspergillus* (Bordin et al., 2014). Several researchers have suggested different strategies to cope with the attack by *Aspergillus* species and avoid aflatoxin contamination (Cobos et al., 2018; Sharma et al., 2018; Khan and Zhuang, 2019). Genetic engineering with *Aspergillus* species-resistant genes is one of the most promising ways to tackle the issue (Nigam et al., 2009; Sharma et al., 2018; Ncube and Maphosa, 2020), especially when no germplasm has found stable resistance to *A. flavus*, which makes breeding resistance variety challenging and very slowly. However, most transgenes are expressed under the control of constitutive promoters that often make plants experience an increased metabolic burden. Constitutive expression of transgenes wastes a lot of metabolic energy that may result in undesirable phenotypes and decreased output (Yuan et al., 2019). Under normal and stressful situations, plants need to transfer important nutrients to the target locations in order to survive and develop smoothly (Divya et al., 2019). As a result, inducible or specific promoters outperform the constitutive promoters to improve the performance of transgene by changing the genetic architecture in an inducible or time- and space-defined manner as plants need. In this study we first found out and characterized the *AhOMT1P* as an *A. flavus* inducible promoter. It shows trace expression under normal conditions but highly upregulated the *OMT1* gene under *A. flavus* infection. Thereby, this provides a new resource for managing aflatoxin contamination in the future peanut enhancement.

Promoters are key regulatory elements that switch on and off the gene functioning. Promoters of *A. flavus* inducible genes can hypothetically drive the expression of a gene upon *A. flavus* infection. Without functional evidence that an inducible promoter can drive a resistance-related gene, we cannot proceed further with the transgenic program. For screening a target promoter, we identified the *AhOMT1* through a genome-wide transcriptomic microarray analysis under drought and a combination of drought and *A. flavus* infection (Chen et al., 2016; Zhang et al., 2017). The microarray expression analysis showed that the *AhOMT1* belonging to peanut methyltransferase family named “Isoliquiritigenin 2’-O-methyltransferase” was highly expressed under drought and *A. flavus* inoculation. It showed a more than 200-fold increase in expression compared to drought control(**Table S1**). The *AhOMT1* gene was cloned from the roots of peanut cultivar Minhua-6 by RACE PCR using a set of specifically designed primers (**Table S2**). The promoter region was also cloned from the roots of the same cultivar systematically. From the available datasets, we identified that it had a trace expression in different tissues or organs, low expression induced by wide environmental stresses like hormones, low temperature, drought, Ca^2+^, and biotic stress like *R. solanacerum* in comparison with the house-keep gene the Actin-7 (**Table S1-1~3**), but a gigantic and specific increase in expression was induced by *A. flavus* infection. We checked the expression of the *AhOMT1* gene in a widely cultivated variety M-6 by qRT-PCR in response to *A. flavus* infection. All these showed the *AhOMT1P* is proper for the anti-aflatoxin purpose. Thus far, a lot of work has been done on the functional characterization of abiotic stress-inducible promoters in crop plants (Pino et al., 2007; Rai et al., 2009; Divya et al., 2019), but there is a lack of work on the functional studies of biotic stress-inducible promoters. Especially it is very hard to find any study reporting *A. flavus* inducible promoters in crop plants.

As the available datasets and expression validation supported that the *AhOMT1* gene is an *A. flavus* inducible gene, its promoter might be a suitable alternative to drive the expression of any foreign gene (related to *A. flavus* resistance) in transgenic plants. To validate its functions, we cloned the promoter of the *AhOMT1* gene and used it to drive the GUS gene under *A. flavus* infection in transgenic plants. The online promoter analysis tools predicted several important *cis*-regulatory elements, including TATA Box, CAAT Box, GC Box (Muthusamy et al., 2017), and many others related to hormones, light, growth, regulation, and stress responsiveness. Different kinds of elements and their number critically determine the strength of a promoter (Stålberg et al., 1993; Tang et al., 2015). Seed-specific elements, including RY-repeats, were also present (Fujiwara and Beachy, 1994). Although these predicted elements are very important for promoter functioning, not a single element related to biotic stress was identified. Perhaps it is due to the reason that not extensive work is available for the binding sites of biotic stress-responsive elements. The promoter analysis also revealed some novel elements (CTCC). It is possible this is a binding sequence for some element involved in Aflatoxin-inducible activity.

Although peanut is much more suitable for functional studies of genes and promoters, but challenging and time-consuming work of genetic transformation makes it less favourite as a model species. In contrast, the short life cycle, easy handling, and well-established genetic transformation methods make *Arabidopsis* a good alternative for functional investigations (Sunkara et al., 2014). So, we choose *Arabidopsis* for the genetic transformation and functional characterization of the *AhOMT1* gene. The results indicated that *AhOMT1* is highly inducible and strongly drives the GUS gene in transgenic plants upon *A. flavus* infection. Although minor deviations in expression patterns were found in response to hormone treatments in transgenic plants, the reason for this deviation can be the different species and another because it was the *GUS* gene under the *AhOMT1* promoter, not the *AhOMT1* gene itself. We are highly convinced that the *AhOMT1P* promoter analyzed here could potentially drive aflatoxin-resistant genes in an inducible manner upon *A. flavus* infection to cope with this serious issue.

## Conclusion

We identified a novel A. flavus inducible gene in this study and worked out its promoter. This gene belonged to peanut O-methyltransferase gene family (Isoliquiritigenin 2’-O-methyltransferase), transferring the methyl group of S-adenosyl-L-methionine (SAM) to the hydroxyl group of numerous different organic chemical compounds, ultimately leading to the synthesis of the methyl ether variants of these substances. This gene was identified by the genome-wide microarray expression under drought and *A. flavus* inoculation. The *AhOMT1* promoter showed trace or no expression in any organs/tissues, nor was it inducible by abiotic or bacterial challenges. Upon infection of *A. flavus* spores, this gene was highly induced in peanut leaves and pericarp. A 1698 bp upstream region of the *AhOMT1* gene was fused with the GUS reporter gene and transformed into *Arabidopsis* plants. Histochemical GUS staining of transgenic *Arabidopsis* plants showed that the *AhOMT1* promoter activated the *GUS* gene only after *A. flavus* infection. While it did not drive the *GUS* expression in normal conditions, nor was it induced by hormone treatments. The real-time expression of the *GUS* gene in transgenic plants confirmed increased expression in response to *A. flavus* infection. These results provided the practical significance of *AhOMT1P* to drive a gene in response to *A. flavus* infection.

## Data availability statement

The original contributions presented in the study are included in the article/**Supplementary Material**. Further inquiries can be directed to the corresponding author.

## Author contributions

Idea was conceived by ZW. ZW, YZ designed the study. YZ, YS investigated the project and wrote the manuscript. XZ, SZC, HC, CZ, YD, MR, and SLC participated in research work, wrote or revised the manuscript. YZ and YS equally contributed to the manuscript. All authors contributed to the article and approved the submitted version.
