# Supplementary material for: Molecular cloning and functional characterization of the promoter of a novel Aspergillus flavus inducible gene (AhOMT1) from peanut
Source: Front Plant Sci. 2023 Feb 9;14:1102181. doi: 10.3389/fpls.2023.1102181 (PMC9947529; doi:10.3389/fpls.2023.1102181)
Supplement: Supplementary file 1 [file DataSheet_1.zip › Supplementay Materials/Supplementary Figures.docx]

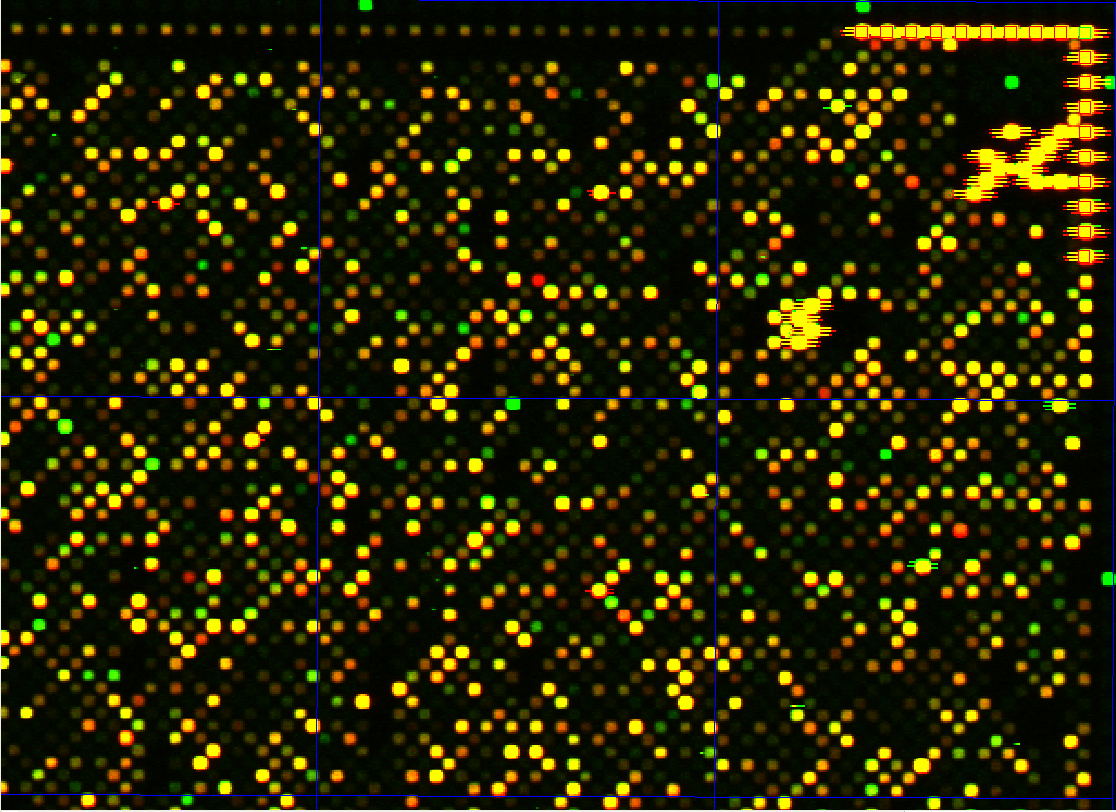


A

B


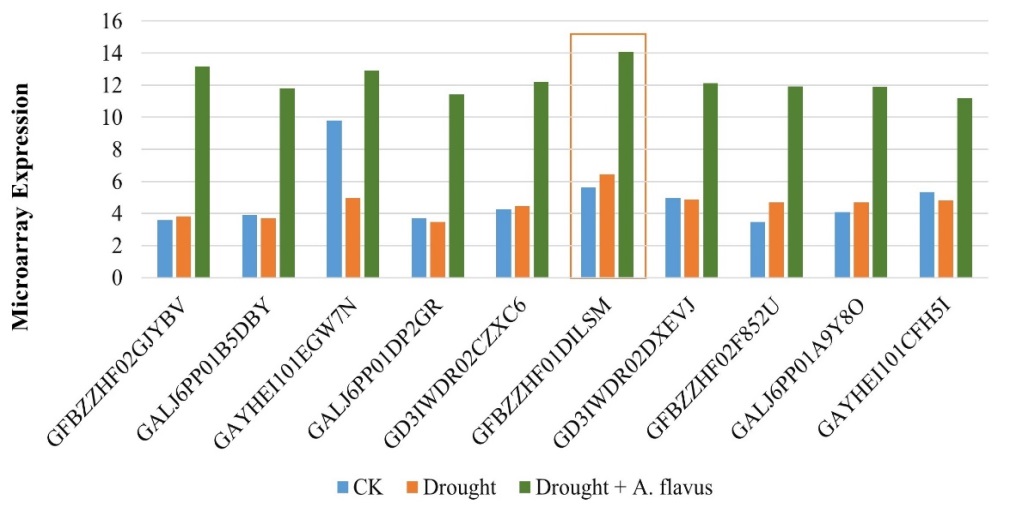


C

**Figure S1. The characterization of *AhOMT1* gene expression.** A. In silic analysis of genes including *AhOMT1* expression was performed on high density of oligo-nucleotide microarray with 100,000 unigenes. B. *AhOMT1* gene (GFBZZHF01DILSM) showed highest expression under a combination of drought and *A. flavus* induction. Log2 normalized microarray expression values of some highly inducible genes are given. C. The expression function of *AhOMT1* across tissues and developing pod.

**1** **GAACACACATTATTATATTCACAAGACAAGTGAGAAAAGAAATTAAACAATGAGTTTGAGCTCCAAAGAGGAAGAAAACAATGCTTTCGT**

**91 TTCAGCATTGGTACTTTGTTTTAGTCGGATCACTCCTGCAATCATAAATGCAGCCATTGATCTGAATCTGTTTGAGATCATACCATCAAA**

**181 AGGGATCATGTCTGCCTCGGAAATTGCTTCAAAGCTTCCGATTAAAAAAGAAGACACTGATATGGTGGCAAAGAGGCTGGAGCGCATGCT**

**271 GCCTTCACTGGCAAGCCACTCGCTTCTAGAATGCTCTATTCGCATTAACCAAGATGGCACCAAAGAAAGAGTTTATGCTCTCTCAAATGT**

**361 TGGTGCCTACTTCAGATCCTCTCGTCATGAACATGAATATGGCGGCTCCTTGACTCCTCTCTCTGTTCTCTTCCATCGAGTATATGATGA**

**451 CCTTTGGTaatctcatttatctaaacacatcatatcatatgccaaatttggtaactgtgaaaatacgtctacgctacatgtacaccaaaa**

**541 tcaactatcaaaaccaaccgccagtatatactatatacacaaaaatattagtaaccgattttagtgtacaaatagtatttttggtaaaaa**

**631 tatgttttctcttagtttttttttttatttaaaccctccacacttgctttttgtttatatagattggaaaaaccattaatcctaggttac**

**721 cacaacacacccacattacacactcattcatacaacactaaactagcgtttgttttgaggtattgagacagagactgaaactcagtatca**

**811 tgtttgttagttcagagactaatactaaaatttctgtctctgttcctaaaacttcagtatttcagtacctccaaaaaatagggacacaag**

**901 ggactaaaatttttagagatggagattgaaactttaataacattttatacctaaaatactttcattttaattaattaatttcaattttat**

**991 cctttgtgcaaattaaattagagtttcattcttgtttcaattcctgtctcccatttttcaccaaacacaatactgagatttattttaatc**

**1081 tctgttttttagtctctgtctctcagtctcagtcttttctctccacgaaacgctacctaaagaattcatattaatattcggcacattcgc**

**1171 caaagttcgaactcaggtgcagcatattaacttattaaggggcatacttaagctagggcctcagctgcacattatttcaagtcttaactt**

**1261 ttcttcttgcAGGAAGGACGCAAAAGATGCAATTTTGGACCCAAATAACAATCATTTTGAAAGGGTTTATGGAATGCCAATGTTCGAATA**

**1351 TTTGAAGACAAACAAAGAAATTAGCCATGTTTTTGACCAAACAATGGCCCAATCTGGCCCACTGGGAATGAAAAGGATCATAAGCCTATA**

**1441 CAAAGGTTTTGAGGGAGTGACAACATTGGTTGATGTAGGAGGTGGAATAGGACAAACCTTAAACTTGATCATTTCTGCGTTTCCTTCCAT**

**1531 TAAAGGAATTAATTTTGATATGCCTCATGTTGTGAAAAACGCACCATCATATCTAGGTatatacacatagtttctgtcaggcttaattat**

**1621 tttatttatagcttttaaattttaataatcaactaaagggcctaattcaatctaatatctactaagtattaacacactaatttttgcata**

**1711 aAGGGATGAGCACGTTGGAGGAGACAGTTCGAAAGTGTTCCAAGGGTGATGCCATTATGCTAAAGGTttggtccctacctgaattcaggc**

**1801 taatttttttttttgttatggaaataaaagtggtgatatactttaatattgtaatttttggtgtttttcaaaattgtagaagtacacaaa**

**1891 ttcctgggtccgatttctgtacttaaaattttttaattttttttaacacaaatccctgggatctatttgtgtacctctcagaaattcctg**

**1981** ggtccaatttgtgtatctctcagaaatcagtcggtccgatttttgtacctctagaaatcggacggtccgatttatgtacctctaacaaat

**2071**  cggacggtccgatttctgcttctctagttaaacgatcccacatttaagtataacaccccaacaatccacatttaagaaaaacaccactaa

**2161 ttccctgaattcatcaaattttttttatacaacataatatatagatgatgtgatatagattttgttttatatttgggtttccAG****CATGTA**

**2251 TGTCATAATTGGTCAGATGAAGAATGCGTCAAATTCCTAAGAAACTGCCACGAAGCGTTGCCACAACATGGAAAGGTGATTGTTTTGGAC**

**2341 TTCATAATGCCAGAAATCCCGAATTCAAGCAATTCATCAAAACATATTTGTGATGTGGACTACCTCATGTTTATAATTAATGGCGGAAAG**

**2431 GAACGAACCCAGAAAGAATTTGAGAGGCTATGCATCAACTCTGGATTTTCTAGATTCCAAGTTGCTTGCACTACTTCCTTAACTACGTTT**

**2521 GCAGTCATAGAATTTTACAAATAAATATTTTGAATATTTTTTTTTCTTTTTTGGTTTTAATTTAATTTGCTCCACCTTCTTGCTTCTTAG**

**2611 CTCTTGTGCACTTTTCTTTTATCTTCGTCACATATCATATATTGTGAGCAATTTACTATTTATGGAATAAGGATTGCGTTTATTTTTTTT**

**2701 TTAAAAAAAAAAAAAAAAAAAAAAAAAAAAAAA**

**Figure S2. The nt sequence of *AOMT1* cDNA and gDNA.** The predicted star codon (ATG) and stop codon (TAA) was underlined.Three introns are in lower case, 816bp, 127bp and 429bp, respectively. The usual 5’GT and 3’AG intron-cut recognize sites was shown in larger font.


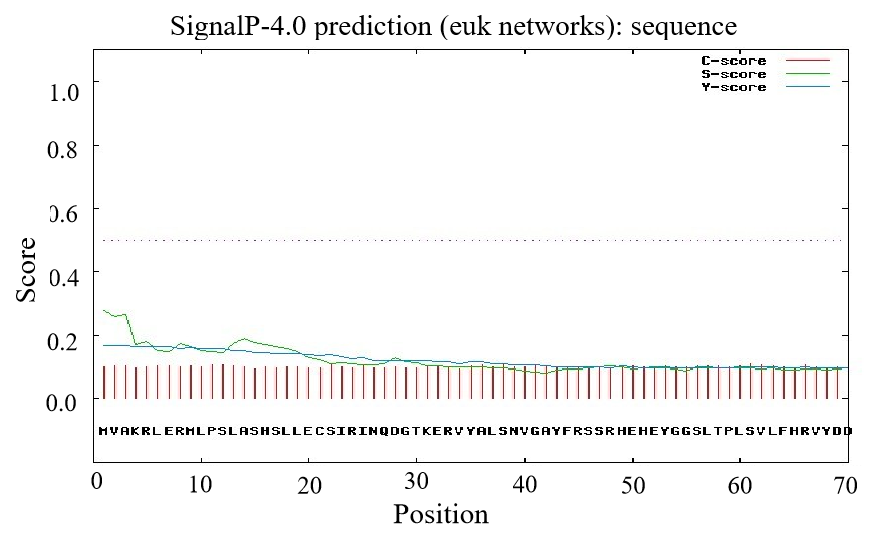

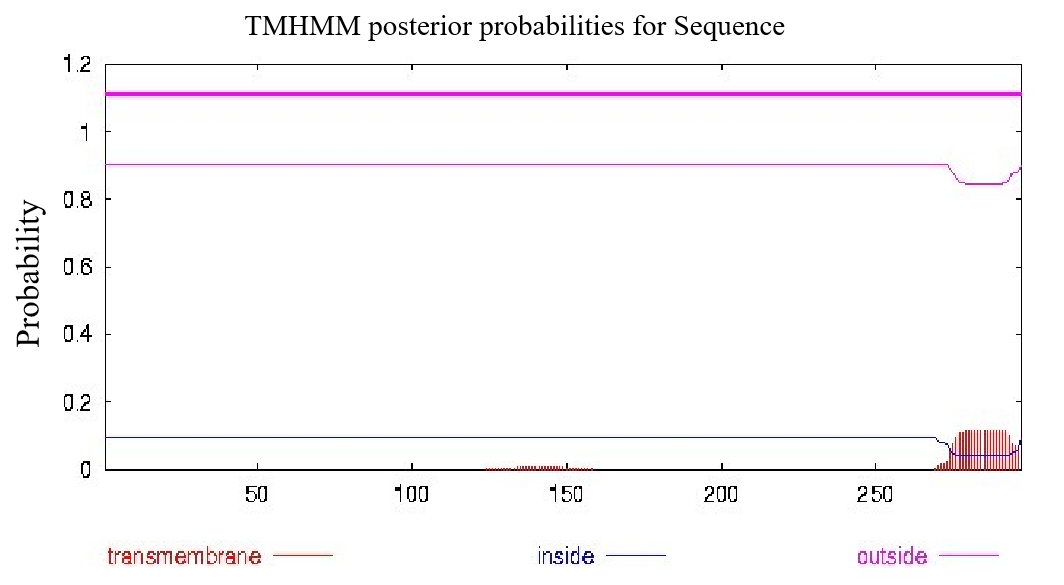

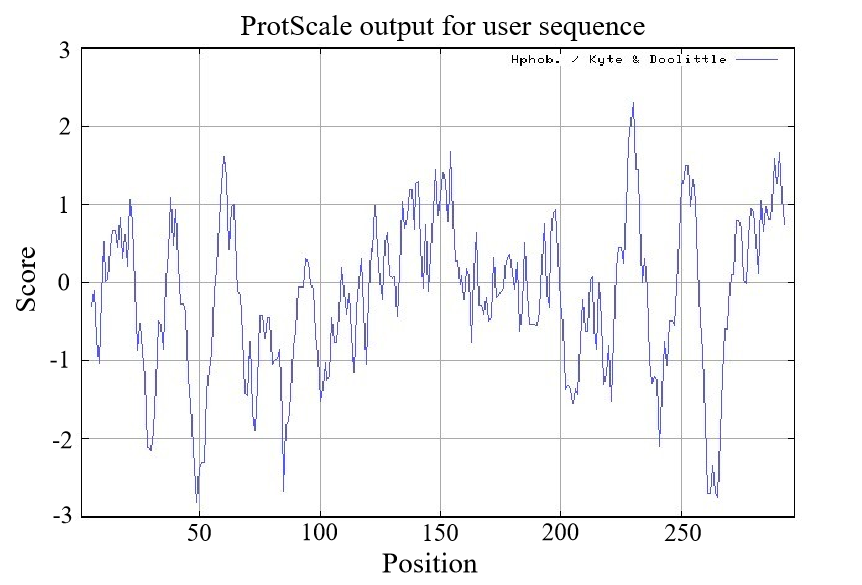

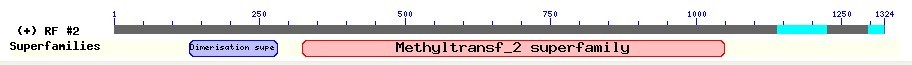

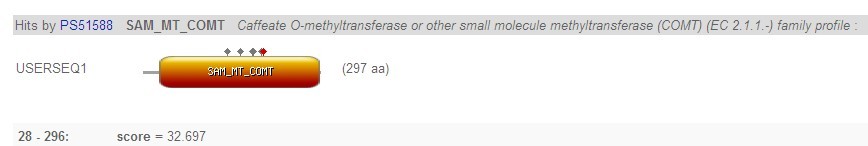


A

E

D

C

B

E

D

**Figure S3. The functional domains analysis of *AhOMT1* gene.** The protein sequences were analyzed by ExPASy and NCBI databases. A. Signal peptide prediction. No signal peptide region was found in this protein. B. Membrane-spanning regions prediction. C. Prediction of protein hydropathicity. The proportion of hydrophilic amino acids was larger than hydrophobic amino acids. D. Protein binding sites. It contains five S-adenosyl-L-methionine binding site and a proton acceptor site. E. The conservative domains. This protein belongs to Methyltransf-2 family. This family includes a range of O-methyltransferases.


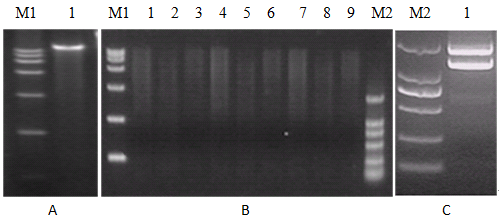


M1 1 M1 1 2 3 4 5 6 7 8 9 M2 M2 1

A

B

C

**Figure S4. The cloning of the promoter of the *AhOMT1* gene.** **A.** The genomic DNA isolated from young leaf of peanut. **B.** Genomic DNA was incompletely digested by the restriction enzyme HindIII, EcoRI and AseI for 5min, 15min and 25min, respectively. **C.** The amplify result of the *AhOMT1* promoter use primer AhAF7-F and AhAF7-R. M1: Marker 15000; M2: Marker2000.

A


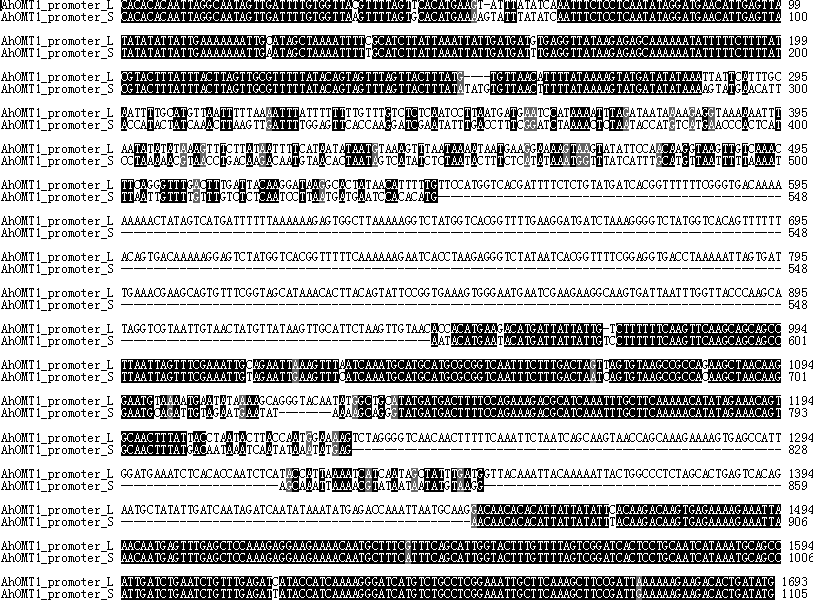


B


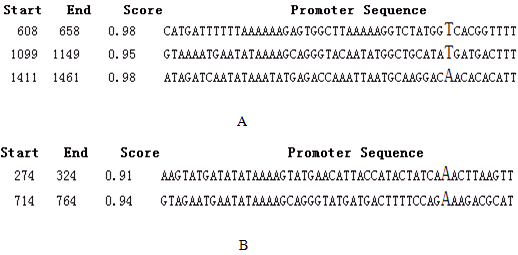


**Figure S5. The sequence and transcription start sites of *AhOMT1* promoter. A.** They were named as AhOMT1_promoter_L and AhOMT1_promoter_S. The promoter. **B.** The transcription starts sites prediction of AhOMT1_promoter_L. They both start at A indicated on the Figure B with high scores.


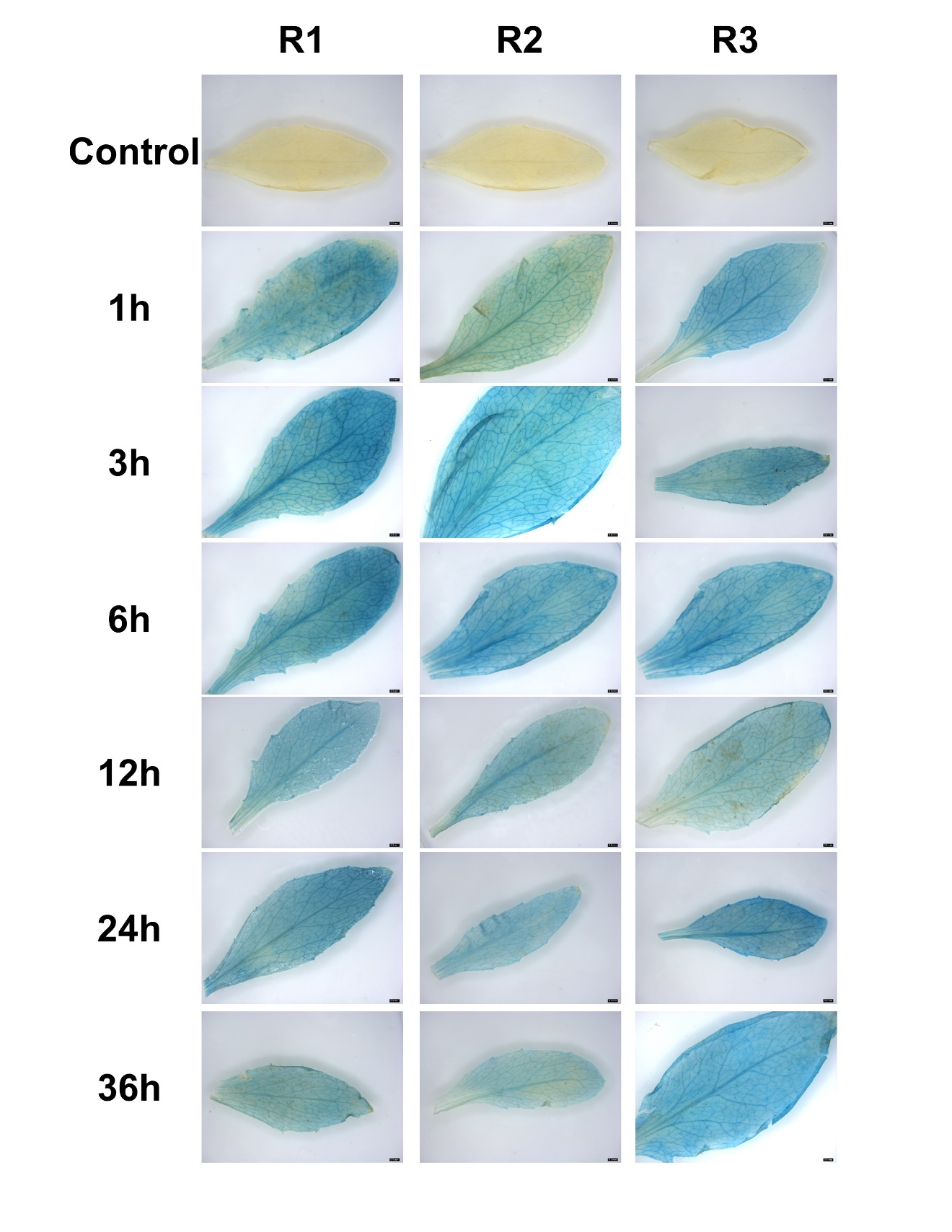


**Figure S6. GUS staining of transgenic Arabidopsis leaves infected with *A. flavus* spores.** The staining results showed that *AhOMT1P* has induced the *GUS* gene in response to spores’ infection. The transgenic control leaves without *A. flavus* inoculation showed not any GUS staining activity, indicating AhOMT1P is *A. flavus* inducible.


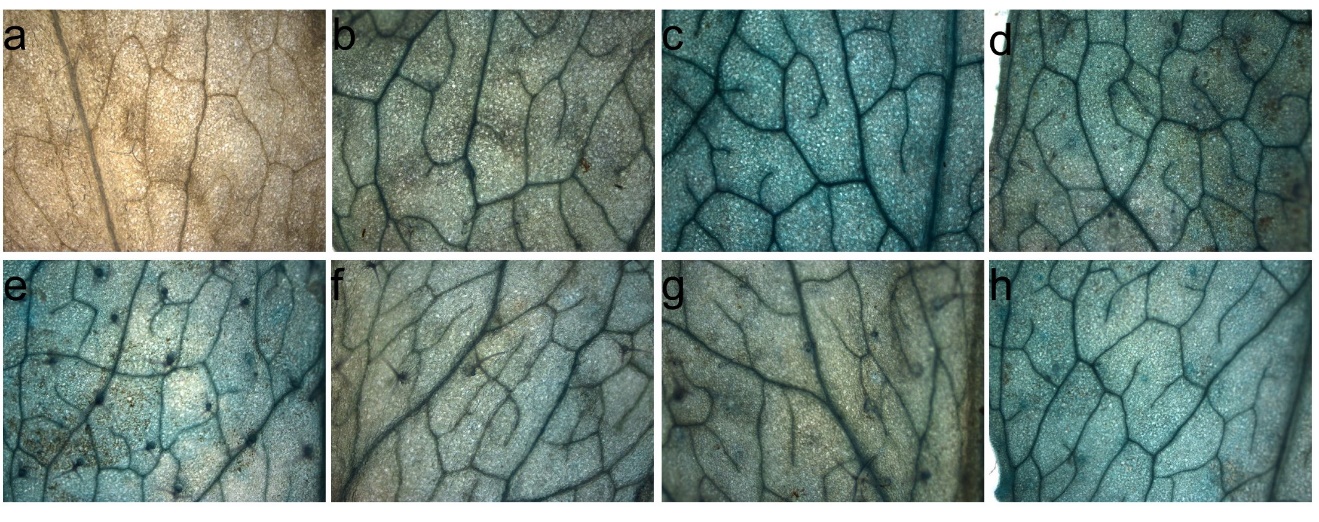


**Figure S7.** **GUS staining of transgenic *Arabidopsis* plants infected with *A. flavus* spores.** Magnified small leaf surface was investigated under the microscope. Here a= control, b= 1h, c = 3h, d = 6h, e = 9h, f = 12h, g = 24h, and h = 36h.


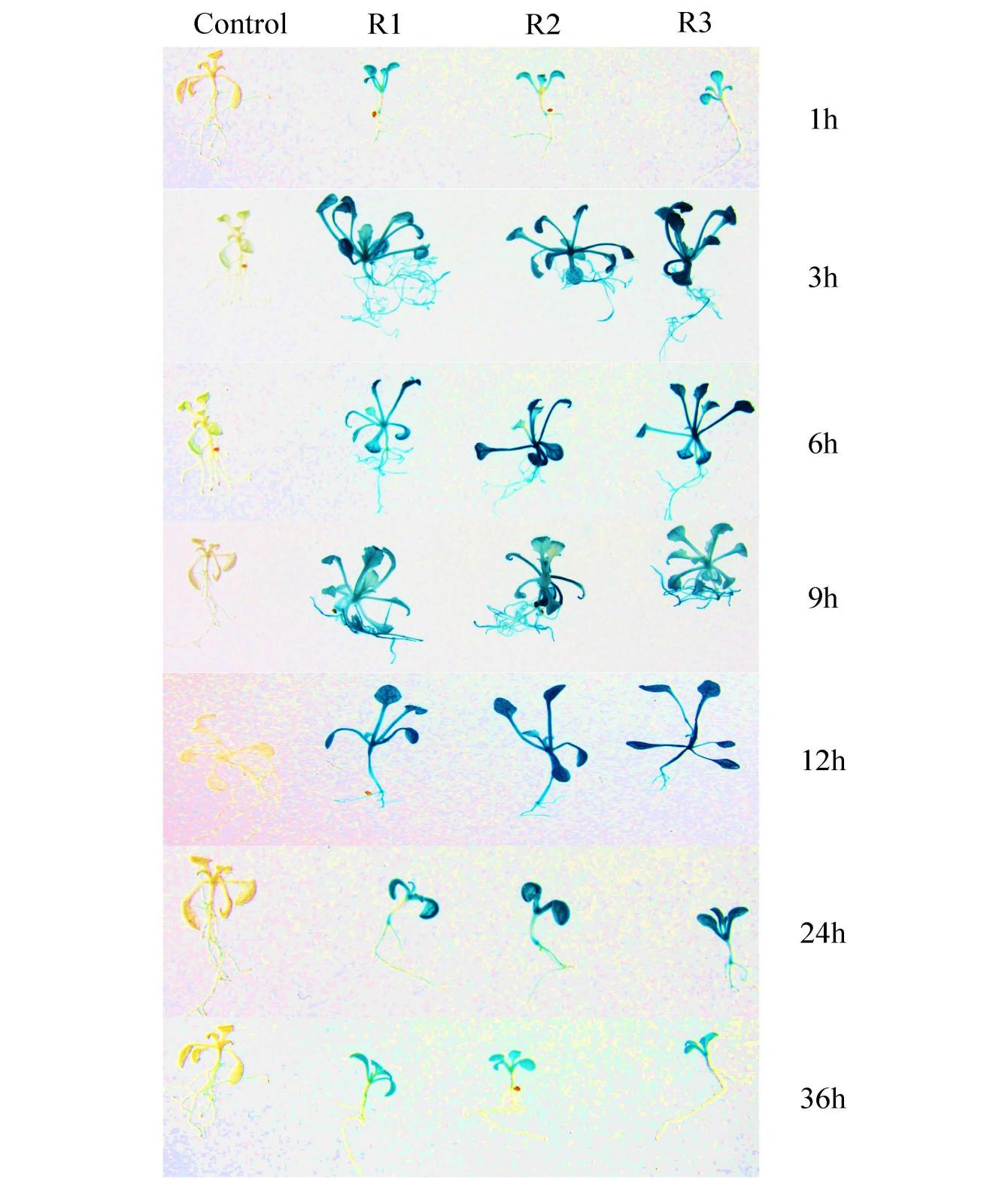


**Figure S8**. GUS staining of transgenic Arabidopsis seedlings infected with *A. flavus* spores. The staining results showed that *AhOMT1P* has induced the *GUS* gene in response to spores’ infection. *A. flavus* spores were used to spray the small seedlings.
