# Supplementary material for: Molecular cloning and functional characterization of the promoter of a novel Aspergillus flavus inducible gene (AhOMT1) from peanut
Source: Front Plant Sci. 2023 Feb 9;14:1102181. doi: 10.3389/fpls.2023.1102181 (PMC9947529; doi:10.3389/fpls.2023.1102181)
Supplement: Supplementary file 1 [file DataSheet_1.zip › Supplementay Materials/Supplementary File 1.docx]

**Supplementary File 1:** Nucleotide sequence of *AhOMT1* promoter

>AH13G54850.1

CGACGATACACACACAATTAGGCAATAGTTGATTTTGTGGTTACGTTTTAGTTCACATGAAGTATTTATATCAAATTTCTCCTCAATATAGGATGAACATTGAGTTATATATATTATTGAAAAAAATTGCATAGCTAAAATTTTCGCATCTTATTAAATTATTGATGATGTGAGGTTATAAGAGAGCAAAAAATATTTTTCTTTTATCGTACTTTATTTACTTAGTTGCGTTTTTATACAGTAGTTTAGTTACTTTATGTGTTAACATTTTATAAAAGTATGATATATAAATTATTCATTTGCAATTTTGCATGTTAATTTTTAAAATTTATTTTTTTTGTTTGTCTCTCAATCCTTAATGATGAATCCATAAAATTTAGATAATAAAAGAGGTAAAAAATTTAATATATATAAAGTTTCTTATAATTTTCATAATATAATGTAAAGTTTAATAAAATAATGAAGGAAAAGTAAGTATATTCCAACAAGGTAAGTTGTCAAACTTCAGGGTTTGACTTTGATTACAAGGATAAGGCACTATAACATTTTTGTTCCATGGTCACGATTTTCTCTGTATGATCACGGTTTTTTAGGGTGACAAAAAAAAAACTATAGTCATGATTTTTTAAAAAAGAGTGGCTTAAAAAGGTCTATGGTCACGGTTTTGAAGGATGATCTAAAGGGGTCTATGGTCACAGTTTTTTACAGTGACAAAAAAGAGTCTATGGTCACGGTTTTTCAAAAAAGAATCACCTAAGAGGGTCTATAATCACGGTTTTCGGAGGTGACCTAAAAATTAGTGATTGAAACGAAGCAGTGTTTCGGTAGCATAAACACTTACAGTATTCCGGTGAAAGTGGGAATGAATCGAAGAAGGCAAGTGATTAATTTAGTTACCCAAGCATAGGTCGTAATTGTAACTATGTTATAAGTTGCATTCTAAGTTGTAACACCACATGAAGACATGATTATTATTGTCTTTTTTCAAGTTCAAGCAGCAGCCTTAATTAGTTTCGAAATTGCAGAATTAAAGTTTAATCAAATGCATGCATGCGCGGTCAATTTCTTTGACTAGTTAGTGTAAGCCGCCAGAAGCTAACAAGGAATGTAAAATGAATATAAAAGCAGGGTACAATATGGCTGCATATGATGACTTTTCCAGAAAGACGCATCAAATTTGCTTCAAAAACATATAGAAACAGTGCAACTTTATTACCTAATACTTACCAATGGAAAAGTCTAGGGGTCAACAACTTTTTCAAATTCTAATCAGCAAGTAACCAGCAAAGAAAAGTGAGCCATTGGATGAAATCTCACACCAATCTCATACCATTAAAATCATCATTAATAGCTATTTGATGGTTACAAATTACAAAAATTACTGGCCCTCTAGCACTGAGTCATCAGAATGCTATGACAATAGATCAATATAAATATGAGACCAAATTAATGCAAGGAC*A*ACACACATTATTATATTCACAAGACAAGTGAGAAAAGAAATTAAACA***ATG***AGTTTGAGCTCCAAAGAGGAAGAAAACAATGCTTTCGTTTCAGCATTGGTACTTTGTTTTAGTCGGATCACTCCTGCAATCATAAATGCAGCCATTGATCTGAATCTGTTTGAGATCATACCATCAAAAGGGATCATGTCTGCCTCGGAAATTGCTTCAAAGCTTCCGATTAAAAAAGAAGACACTG**ATG**GTGGCAAAGAGGCTGG
