# Supplementary material for: Molecular cloning and functional characterization of the promoter of a novel Aspergillus flavus inducible gene (AhOMT1) from peanut
Source: Front Plant Sci. 2023 Feb 9;14:1102181. doi: 10.3389/fpls.2023.1102181 (PMC9947529; doi:10.3389/fpls.2023.1102181)
Supplement: Supplementary file 1 [file DataSheet_1.zip › Supplementay Materials/Table S2.docx]

**Table S2. Primers used in studying *AhOMT1* promoter**

| **qRT-PCR** | |
| --- | --- |
| AhActin-F | GAGGAGAATCAGAAGCAAGTC |
| AhActin-R | CATATACAGCATAGCGGCACTC |
| q-AhOMT1-F | ATACCATCAAAAGGGATCATGTCT |
| q-AhOMT1-R | ATAAACTCTTTCTTTGGTGCCATC |
| **RACE cloning *AhOMT1* gene: 5' fragment and 3' fragment, respectively** | |
| RACE-F | 5’-CAGTAGATCTGTAATACGACTCAC-3’ |
| AhOMT1-R | 5’-TGGTTAATGCGAATAGAGCATTC-3’ |
| RACE-R | 5’AAGCAGTGGTATCAACGCAGAGTGGCCGAGGCGGCCGATTTTTTTTTTTTTTTTTTTTTTTTTTTTTTVN-3' |
| AhOMT1-F | 5’-TCGTTTCAGCATTGGTACTTTG’ |
| **Primers for promoter cloning by Flanking PCR** | |
| OngAd | 5’-GTAATACGACTCACTATAGGGCACGCGTGGTCGACGGCCCGGGCTGC-3’ |
| ShortHindIII | 5’-P-AGCTGCAGCCCG-NH2-3’ |
| ShortEcoRI | 5’-P-AATTGCAGCCCG-NH2-3’ |
| ShortAseI | 5’-P-TAGCAGCCCG-NH2-3’ |
| Adapter Primer (AP1) | 5'-GTA ATA CGA CTC ACT ATA GGGC-3' |
| Adapter Primer (AP2-C) | 5'-TGG TCG ACG GCC CGG GCT GC-3' |
| AhOMT1-SP1 | 5’- AGACATGATCCCTTTTGATGGTATAATCTC -3’ |
| AhOMT1-SP2 | 5'- CTGCATTTATGATTGCAGGAGTGATCC -3' |
| AhAF7-F | 5’-CACACACAATTAGGCAATAG-3’ |
| AhAF7-R | 5’-CGATGGAAGAGAACAGAGAG-3’ |
| **Promoter amplification** | |
| AhOMT1P-F | CGACGATTCACACACAATTAGG |
| AhOMT1P-R | CCAGCCTCTTTGCCACCATATC |
| **Promoter Gateway cloning** | |
| GWOMT1-F | GGGGACAAGTTTGTACAAAAAAGCAGGCTTCCGACGATTCACACACAATTAGG |
| GWOMT1-R | GGGGACCACTTTGTACAAGAAAGCTGGGTCCCAGCCTCTTTGCCACCATATC |
| **Transgenic plants confirmation** | |
| OMT-GUS-F | AATCAGCAAGTAACCAGCAAAG |
| OMT-GUS-R | CGGCAATAACATACGGCGTGAC |
